# Diagnostic efficacy of systemic immune-inflammation biomarkers in benign prostatic hyperplasia using receiver operating characteristic and artificial neural network

**DOI:** 10.1038/s41598-023-41781-3

**Published:** 2023-09-08

**Authors:** Rasha Ahmed, Omnia Hamdy, Refaat Mostafa Awad

**Affiliations:** 1 Urology Department, Faculty of Medicine for Girls, Al Azhar University, Cairo, Egypt; 2 Engineering Applications of Lasers Department, National Institute of Laser Enhanced Sciences, Cairo University, Giza, Egypt

**Keywords:** Medical research, Urology

## Abstract

Benign prostatic hyperplasia (BPH) is a chronic, progressive disease characterized by mesenchymal cell-predominance and stromal and glandular cell-hyperproliferation. Although, the precise cause of BPH is unknown, it is believed to be associated with hormonal changes in aging men. Despite androgens and ageing are likely to play a role in the development of BPH, the pathophysiology of BPH remains uncertain. This paper aims to evaluate the diagnostic efficacy of platelet-to-lymphocyte ratio (PLR), neutrophil–lymphocyte ratio (NLR) and systemic immune-inflammation index in in diagnosing BPH. A single-center-randomized-retrospective study was carried out at Alzahraa university hospital between January 2022 and November 2022 on 80 participants (40 non-BPH subjects and 40 patients with symptomatic enlarged prostate) who visited the outpatient clinic or admitted to the urology department. The BPH cases were evaluated by digital rectal examination (DRE), International Prostate Symptom Score (IPSS), prostate size, prostate specific antigen (PSA), TRUS biopsy in elevated PSA > 4 ng/ml, PLR, NLR and systemic immune inflammatory (SII). The diagnosing efficiency of the selected parameters was evaluated using Receiver Operating Characteristic (ROC) and Artificial Neural Network (ANN) showing excellent discrimination with 100% accuracy and AUC = 1 in the ROC curves. Moreover, the accuracy rate of the ANN exceeds 99%. Conclusion: PLR, NLR and SII can be significantly employed for diagnosing BPH.

## Introduction

The medical term for an enlarged prostate that can affect how men urine is benign prostatic hyperplasia (BPH)^1^. Although the specific cause of BPH is unknown, it is believed to be related to hormonal changes that occur in men as they age. BPH is a chronic, progressive disease marked by mesenchymal cell predominance and stromal and glandular cells hyperproliferation^2^.

Recent clinical investigations have revealed a connection between prostatic inflammations and lower urinary tract symptoms (LUTS) associated with BPH^3,4^, and over the past few years, it has become clear that inflammation may play a significant role in the development and progression of BPH^5,6^. Consequently, it has been proposed that BPH is an inflammatory immune-mediated condition, and that inflammation may directly influence prostate growth^7,8^. The autonomic nervous system regulates the adult prostate gland's physiological processes, while hormones control the gland's growth and development. The hypogastric nerve sends sympathetic signals to the prostate gland, whereas the pelvic nerve sends parasympathetic signals. Additionally, the gland receives sensory input from the pelvic and hypogastric nerves^9^.

A typical diagnostic test for detecting BPH is a blood test that measures the amount of prostate-specific antigen (PSA). PSA is a protein generated by both malignant and noncancerous tissue in the prostate^10^. Although it is a substance synthesized in the prostate, men with larger prostates have higher PSA levels. High PSA levels are also thought to be a sign of an enlarged or inflammatory prostate. However, recent treatments, infections, surgeries, or prostate cancer are also possible causes of increased PSA scores^11^. The systemic immune-inflammation index (SII), which depends on the peripheral platelet, neutrophil, and lymphocyte counts, has been proven to be a potential prognostic predictor in a variety of conditions, including tumors, nervous system diseases, chronic heart failure, and prostate cancer^12–14^. Záhorec introduced the NLR to provide a fast detectable parameter for measuring the intensity of stress and systemic inflammation in intensive care patients with shock, numerous traumas, major surgery, or infection^15^. The NLR was earlier thought to be a marker for inflammatory disorders, but nowadays, other conditions are also recognized to change the NLR^16,17^. It has been proven to be a reliable indicator in assessing the diagnostic and therapeutic outcomes for many benign and malignant disorders^18–20^.

Currently, the intriguing NLR argument is covered in several urologic publications. It has been demonstrated that it reflects how much systemic inflammation is involved in neoplasia^21^. Prior to the first conservative natural orifice retrograde endoscopic upper tract urothelial carcinoma (UTUC) evaluation and treatment, a baseline NLR assessment may offer useful, easily accessible, reasonably priced, and objective prognostic information regarding the risk of upper tract but not bladder recurrence. It supplemented high-grade cytology and could one day aid in the individualization of management strategies. If these particular cases result in a more pronounced host inflammatory response, NLR may successfully identify them promptly, allowing earlier categorization for a more aggressive care and surveillance strategy/truly personalized medicine^22^.

Moreover, it was revealed that obese patients exhibited more symptoms than normal-weight patients, specifically a greater storage IPSS score. In patients with benign prostatic hyperplasia and lower urinary tract symptoms (BPH/LUTS) treated with transurethral resection of the prostate (TURP), it was recently discovered that there was a high correlation between prostatic inflammation, defined as an inflammatory score of 7, and an autophagy deficiency. The link between obesity and prostatic inflammatory infiltrates has been established, and obese patients with LUTS/BPH have shown the first signs of autophagy dysregulation. These findings highlight a number of issues regarding the processes underlying the failure of the autophagy, and preliminary indications point to autophagy dysregulation as one potential route connecting inflammation and obesity in BPH/LUTS patients^23^.

There is also evidence that some markers of systemic inflammation have been associated with prostate cancer (PCa). A number of inflammation markers, including C-reactive protein (CRP), neutrophil, lymphocyte, monocyte, platelet, and PLR counts as well as a number of biochemical markers, such as the De Ritis ratio, have been studied in relation to PCa. Clinically severe PCa is far less common in people with gray-zone PSA readings. Combinations of various biochemical parameters are being researched to increase the likelihood of detecting PCa before a biopsy in order to prevent pointless biopsies. In males with gray-zone PSA values, some biochemical metrics, such as CRP, the CRP/albumin ratio, and the De Ritis ratio, may be useful in addition to established markers like PSA, PSAD, and the fPSA/tPSA ratio. CRP is a predictive indicator for PCa patients' overall survival, cancer-specific survival, and progression-free survival. When compared to the BPH group, the PCa group had significantly greater CRP and a higher CRP/albumin ratio^24^.

Additionally, ultrasound (US) is a prevalent and well-tolerated imaging tool for examining the prostate. Recent technological advancements in US applications have created new features of prostate analysis. The use of structural analysis facilitates the measurement of prostate volume, the investigation of echotexture, and the demonstration of tissue stiffness or elasticity. In functional analysis, macrovascularity and microvascularity are depicted as tissue perfusion markers^25^. The availability of portable, light-weight probes, the relative simplicity of learning the fundamentals of the transrectal sonographic prostate biopsy technique, and the ability to image the gland with accurate volumetric measurements have all contributed to the widespread clinical use of transrectal ultrasonography (TRUS), primarily because an alarmingly high proportion of men are getting prostate biopsies after PSA tests. Although TRUS is useful for imaging the prostate and measuring its size, its diagnostic precision for locating cancerous areas is still insufficient^26^.

The responses to seven questions about urine symptoms (intermittency, nocturia, frequency, urgency, straining, incomplete empting, and weak stream) plus one question about quality of life are used to calculate the International Prostate Symptom Score (I-PSS). For each enquiry on urinary symptoms, the patient can choose one of six possibilities, with the options signifying the intensity of the specific ailment. The responses are graded on a scale of 0 to 5. As a result, the overall score can range between 0 (i.e., asymptomatic) and 35 (i.e., very symptomatic)^27^. In the present work, we provide a single-center randomized retrospective descriptive study that pioneers the use of SII, NLR, and PLR as BPH disease indicators. Utilizing Receiver Operating Characteristic (ROC) and Artificial Neural Networks (ANN), the efficacy of employing such factors for diagnosis was evaluated and analogized with the effectiveness of using the IPSS score, prostate size, and prostate-specific antigen (PSA). Based on ROC curve parameters, we primarily assessed the ability of each measure (IPSS, prostate size, PSA, NLR, PLR, and SII) to detect the disease (i.e., BPH). Furthermore, detection efficiency using PLR, NLR, and SII levels was correlated with IPSS severity, as well as the prostate size and PSA score. The three systemic immune-inflammation biomarkers (NLR, PLR, and SII) were then used in the ANN technique to confirm their effectiveness in identifying BPH.

## Methods

After receiving approval from the ethical committee, data from 80 participants (40 patients with symptomatic enlarged prostate and 40 non-BPH individuals as a control group) was retrospectively reviewed in the study. Non-PBH (control) group are individuals with no lower urinary tract symptoms (irritative or obstructive), normal US, normal DRE and zero IPSS. BPH group are patients who have any degree of IPSS and enlarged prostate with no suspicious of malignancy examined by US, DRE or TRUS. PSA was done if it was high with no other reason of elevation. TRUS biopsy was necessary to exclude prostate cancer. If there was suspicious, of prostate cancer by DRE, TRUS biopsy was performed to exclude malignancy. If the patient showed to has prostate cancer by TRUS, he was excluded from the study.

The data was gathered between January and November of 2022. Personal and medical histories of all BPH patients, physical and general examination were noted. Additionally, definitive diagnosis of benign enlarged prostate obtained with digital rectal examination, IPSS, abdominal ultrasound (US) and TRUS were achieved via ultrasound scanner BK Medical Ultrasound Scanner (1202 Flex Focus 200 REF: Type 1202), serum PSA and other laboratory work-up (CBC, urine analysis) have been collected. CBC and PSA were performed using a Sysmex XP-300 and IMMULITE1000 devices respectively. Systemic comorbidities, drug/alcohol abuse; nonsteroidal anti-inflammatory drug use within the last week; upper respiratory infection within 1 month pregnancy; insufficient history information on the evaluation, sepsis, urinary infection, stricture urethra, cancer, and urinary or gall bladder stones were exclusion criterion.

**Figure 1:**
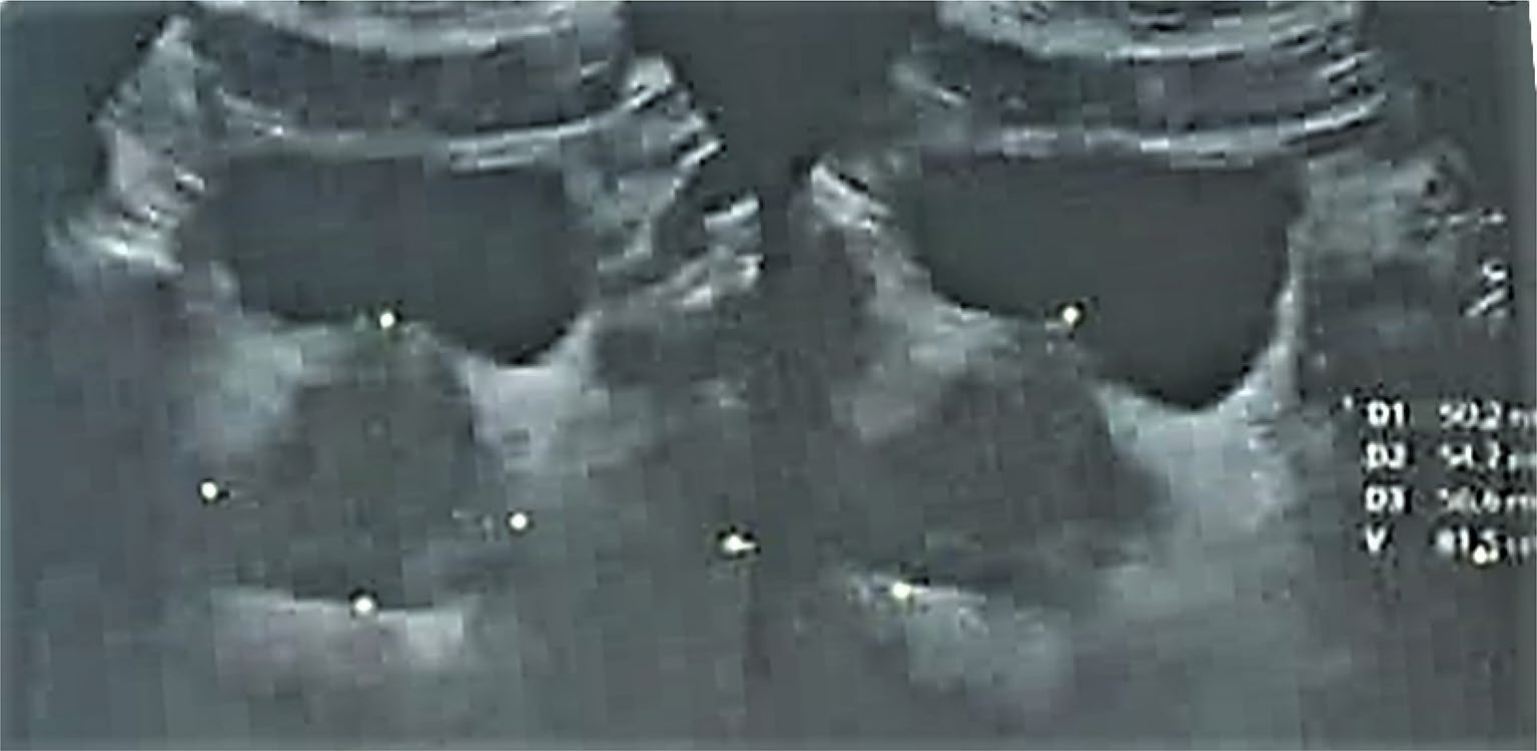
US image of a sample of BPH subject involved in the study.

For TRUS examination, a digital rectal exam must be carried out before the ultrasound probe is inserted. With their legs bent towards their chest, the patient is positioned in the lateral decubitus posture, most frequently on their left side. Prior to this, the lithotomy position was the norm. However, the patient can endure lateral decubitus better. A cover and good lubrication are on the transducer. Following the rectal examination, the transducer is implanted with the ultrasound beam pointed anteriorly towards the patient. To prevent the change of the prostate gland's shape, the least amount of pressure is recommended to be applied on the gland. The symmetry, shape, width of the gland, and related seminal vesicles are all evaluated. The prostate is then measured in the sagittal plane to determine its height, length, seminal vesicles, and prostatic urethra. The evaluation's main factors are the changes which may occur in echogenicity and the overall echogenicity^28^. In the transitional zone, benign prostatic hyperplasia manifests as many hypoechoic nodular regions. From a largely homogeneous to a diverse echogenicity may develop. The peripheral and central zones may begin to compress as the transitional zone grows, making them echogenic.. They become flat and are referred to as the "surgical capsule," which marks the boundary between the hyperplastic and regular peripheral prostatic tissue. When considering BPH, it is critical to assess the prostate's overall volume. The most popular formula for calculating an ellipsoid's volume is height (H) × width (W) × length (L) × π/6. When the volume is larger than 25 cm^3^, the prostate is considered enlarged^26,29^. A sample US image of a BPH patient is provided in Fig. 1.

### Samples collection

A single center randomized retrospective study was carried out at urology department, Alzahraa university hospital between January and November 2022 on 80 individuals (40 non-BPH and 40 BPH patients). All participants provided a written informed consent for all clinical procedures. Each participant was evaluated by the International Prostate Symptom Score (IPSS), prostate size evaluated by ultrasound, prostate specific antigen (PSA), TRUS biopsy in elevated PSA, platelet/lymphocyte (PLR), neutrophil//lymphocyte (NLR) and systemic immune inflammatory (SII). The absolute neutrophil count was divided by the absolute lymphocyte count to determine the NLR. While, the platelet count was divided by the absolute lymphocyte count to calculate the PLR values. Finally, the SII was calculated based on the following equation^12^:

1 $$\mathrm{SII}=\frac{\mathrm{neutrophil\,count}\times \mathrm{ platelet\,count }}{\mathrm{lymphocyte\,count}}$$

All the collected measurements have been analyzed using IBM SPSS Statistics 6 software package. The data was checked for linearity “Supplementary S1” and then comparison was performed in independent groups using “t Test” in SPSS “Supplementary S2”. In general, the overall data analysis provided significant results.

### Receiver operating characteristic (ROC)

ROC is a graphical depiction used to analyze diagnostic test accuracy by visualizing the sensitivity vs. 1-specifity of a certain test or collection of tests^30,31^. The region (i.e., area) underneath the ROC curve (AUC) is regarded as the most important factor in determining the efficacy of the investigated discriminating method (test), as the higher the AUC, the more accurate the test^32,33^. The principle of true positive, true negative, false positive, and false negative is the base for constructing a ROC curve. The Y-axis of the curve depicts sensitivity, which displays the true positive percentage, while the X-axis of the curve reflects 1-specificity, which is the false positive fraction^34^. The sensitivity of the test is defined as Se(p) = 1 − G(p) for two random variables X and Y of distribution functions F and G, respectively, while the specificity is given by Sp(p) = F(p). Accordingly, the ROC curve is constructed by plotting Se(p) versus 1 − Sp(p) for − ∞ ≤ p ≤ ∞.

2 $$ROC\left(t\right)=1-G\left({F}^{-1}\left(1-t\right)\right), over\,t\in [\mathrm{0,1}]$$

3 $$AUC={\int }_{0}^{1}ROC\left(u\right)du$$

In the current study, the exhibited ROC curves were generated using the MATLAB software framework through a proprietary Matlab function. This function plots the ROC curve and calculates its parameters by representing the 1-specificity and sensitivity of two classes of data (i.e., BPH and non-BPH (i.e., control) measures in our implementation).

### Artificial neural networks (ANN)

**Figure 2:**
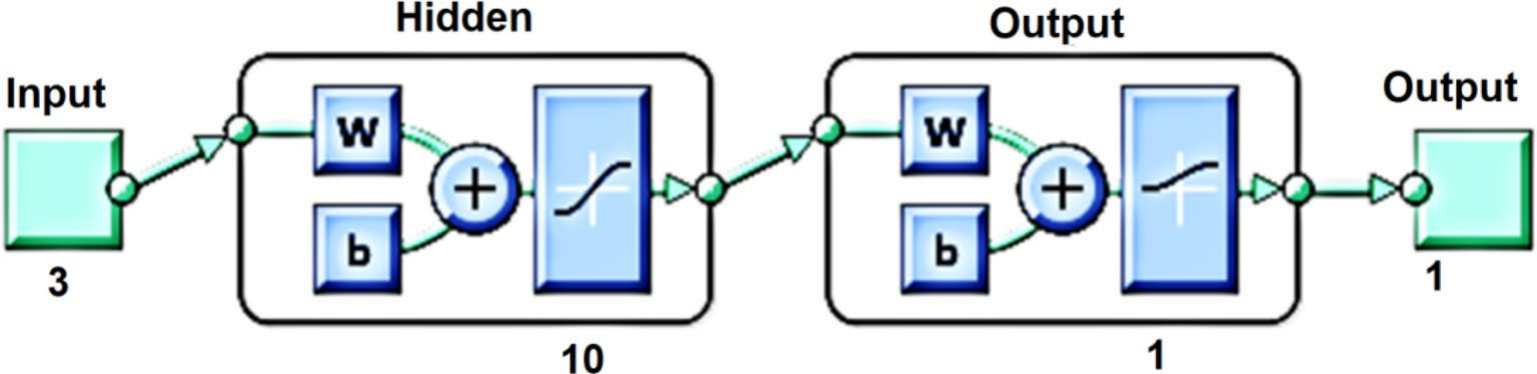
The constructed neural network model.

Inputs are categorized using artificial neural networks based on target classes. The collected measurements for PLR, NLR, and SII have been employed in the current implementation as the three input parameters for a feed-forward network utilizing Matlab software to confirm their efficacy in detecting BPH. A feed-forward network is made up of several levels. The network input is connected to the top layer. There is a connection from the previous layer to each succeeding layer. The output of the network is produced by the top layer. The weight and bias parameters based on the SCG approach have been updated using the network training function "trainscg"^35,36^. Furthermore, 10 neurons were used in the hidden layer of our NN model's implementation. The constructed NN model is shown in Fig. 2.

### Ethics approval and consent to participate

The proposed study was carried out in accordance with the Helsinki Declaration and was approved by the NILES, Cairo University ethics committee (No: NILES-EC-CU 23/1/2). All patients were treated at an academic hospital. All patients were requested to give their general agreement for the scientific examination of disease-specific data in anonymized form upon admission to the hospital. Patients could potentially decline to give their consent.

## Results

### ROC results

**Figure 3:**
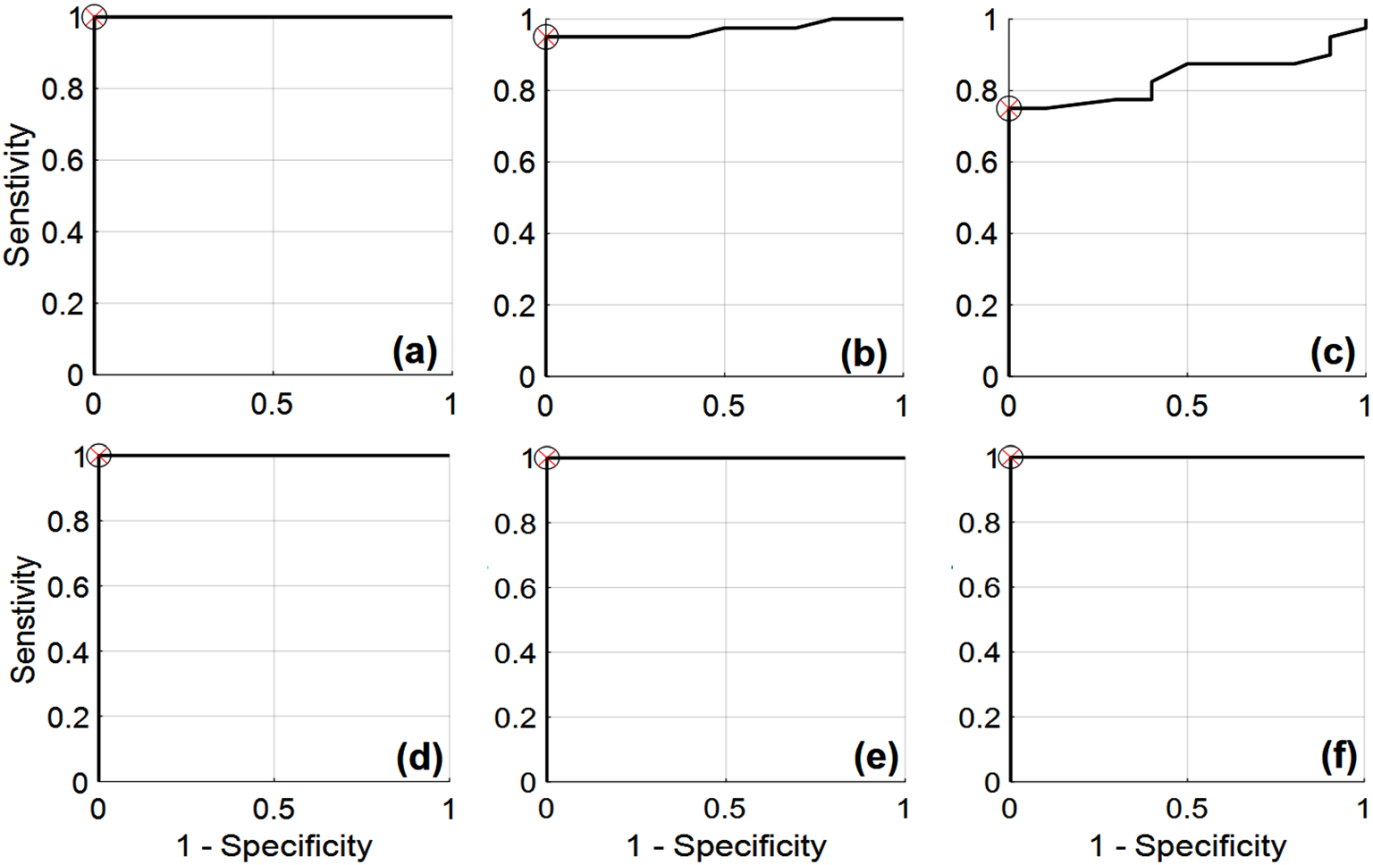
ROC curves between control and BPH data based on the different monitored symptoms, (**a**) IPSS, (**b**) size, (**c**) PSA, (**d**) PLR, (**e**) NLR, and (**f**) SII.

Eighty participants (40 non-BPH and 40 BPH patients) were included in this study with a mean age (~ 66), ranged from (53–83) years. Level of neutrophil, lymphocyte, platelet, and SII are measured. Accordingly, the mean SII values were (6.3726 × 10^5^) with a cutoff value (4.150358 × 10^5^), mean NLR (2.31) with a cutoff value (1.83), and mean PLR (132.86) with a cutoff value (93.58). For each monitored symptom, ROC curve has been created in order to assess the accuracy of the data as illustrated in Fig. 3. Moreover, Table 1 provides a summary of the various ROC-curve metrics, including sensitivity, specificity, and accuracy.

**Table 1:** The obtained ROC curve parameters.

Symptoms	Sensitivity (%)	Specificity (%)	AUC	Accuracy (%)
IPSS	100	100	1	100
Size	95	100	0.97	97.5
PSA	75	100	0.83	87.5
PLR	100	100	1	100
NLR	100	100	1	100
SII	100	100	1	100

ROC analysis revealed a statistically significant correlation between SII, NLR, PLR in identifying BPH disease which was equivalent to IPSS. The sensitivity, accuracy and specificity for each was 100%, and AUC = 1 (see Fig. 2). Using IPSS which was defined as the need for intervention, ROC analysis was performed to compare it with corresponding NLR, PLR and SII values. Although size and PSA measurements show good results as provided in the ROC curves, the optimum discrimination are achieved using IPSS, NLR, PLR and SII values. Additionally, the highest accuracy, sensitivity, specificity and AUC were recorded at these parameters (see Table 1). Accordingly, the proposed ROC parameters reveal excellent discrimination based on NLR, PLR and SII values as suggested in this study.

**Figure 4:**
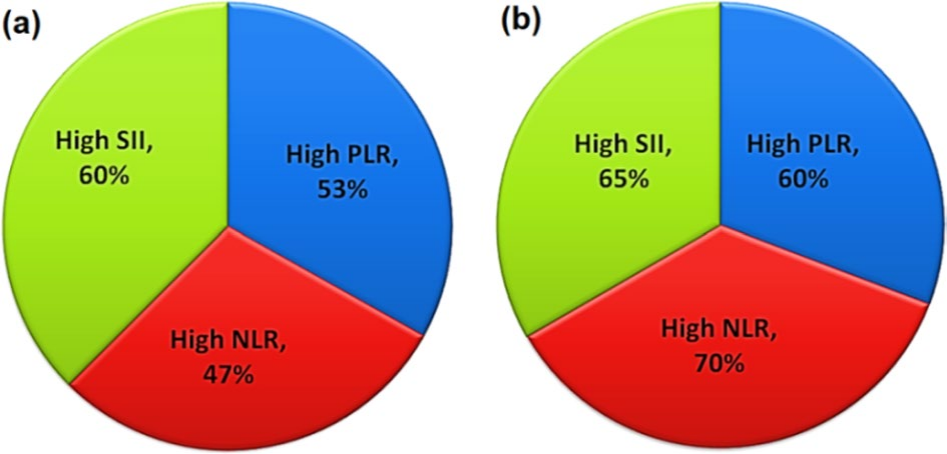
Patient distribution according to their PLR, NLR and SII values (**a**) moderate IPSS, (**b**) severe IPSS.

Based on the optimal cut-off point with the highest sensitivity and specificity for each considered parameter, the examined samples were classified with respect to the IPSS score in comparison with the corresponding NLR, PLR and SII values. In total 23 patients with severe IPSS, 70% has high NLR values, 65% has high SII values and 60% of them have high PLR values. On the other hand, in total 15 patients with moderate IPSS, 47% has high NLR values, 60% has high SII values and 53% of them have high PLR values as illustrated in Fig. 4.

**Figure 5:**
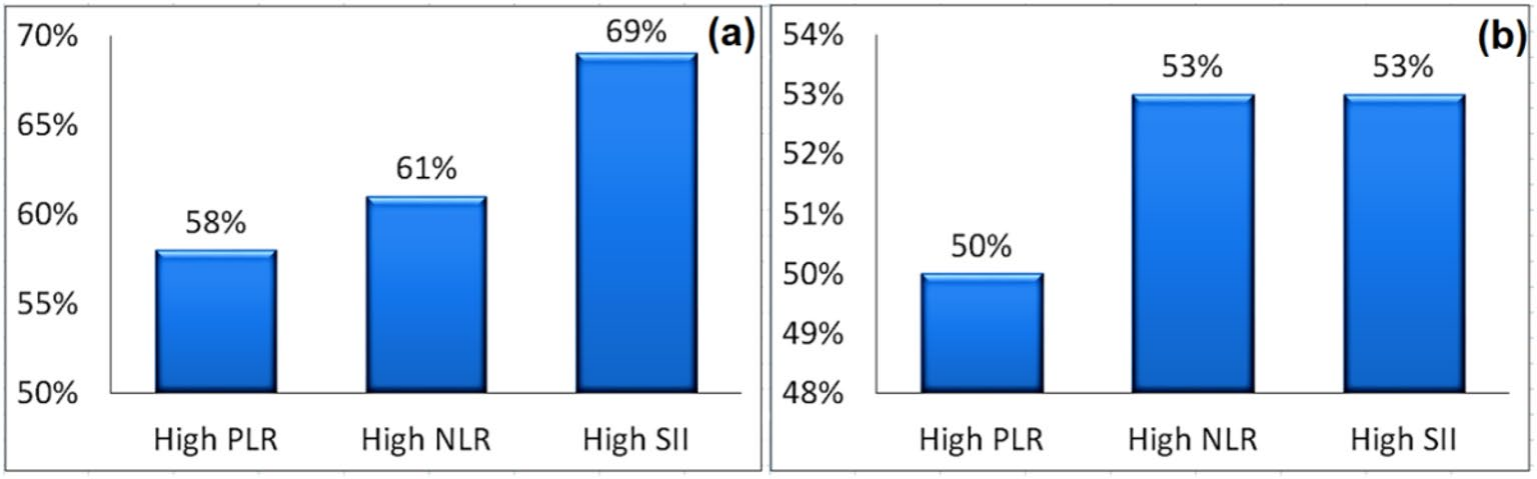
Patient distribution according to their PLR, NLR and SII values (**a**) enlarged size, (**b**) high PSA.

Additionally, both of prostate size and PSA level accompanying NLR, PLR, and SII values were compared. As seen in Fig. 5a, for enlarged prostate size patients, 69% had high SII values, 61% of them showed higher NLR, and 58% exposed high PLR. Additionally, 53% of patients with PSA also had high SII values and high NLR levels; whereas only 50% had high PLR levels (see Fig. 5b).

### ANN results

**Figure 6:**
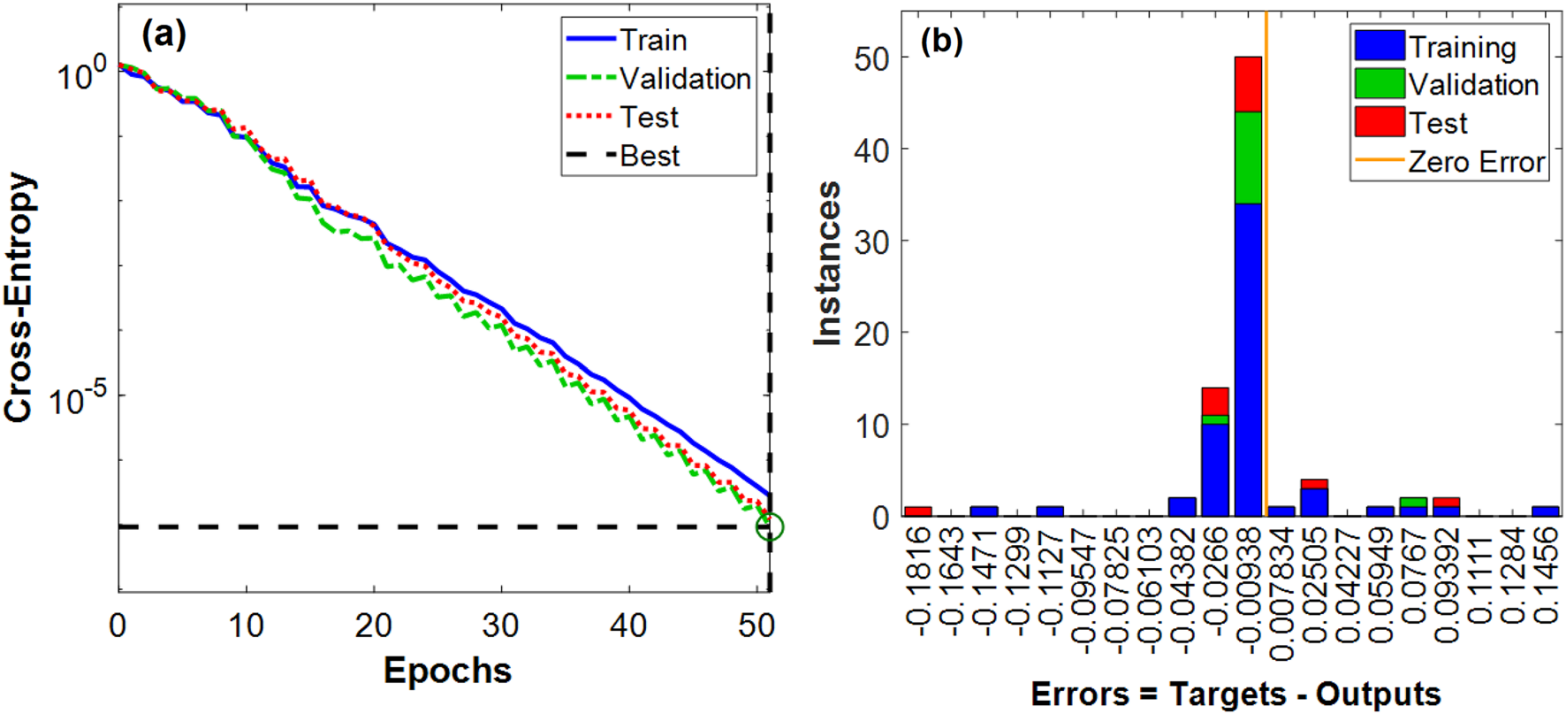
(**a**) Performance plot for the ANN model with best mean square error, (**b**) Error histogram plot for one of the ANN executions.

Several network runs were performed to achieve the best performance. The model with the lowest mean square errors (MSE) performance plot is presented in Fig. 6a. As the number of iterations goes up, the MSE decreases, as seen by the performance curve. The plot further assesses that the features of the error in the validation and testing data sets are nearly identical, and that the model performed at its best during the chosen number of iterations (epochs). Figure 6b provides an example of the error histogram derived from the implemented NN models. The error shows the discrepancy between the desired and actual output that the network produces. The number of samples from the training, validation, and test sets is shown by the term "Instances" in the figure. The figure clearly shows that the distribution of the fitting data errors is close to the zero-error zone, which verifies appropriate operation.

**Figure 7:**
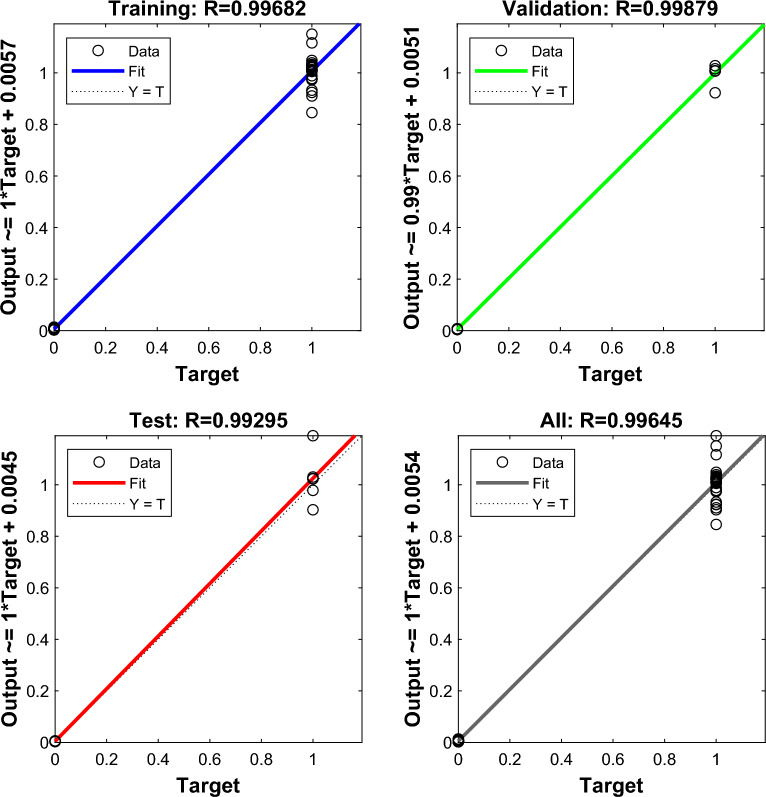
Regression plot for ANN model.

Figure 7 shows the regression plot of correlation coefficient (R) which presents the relationship connection between the outputs and targets for ANN model. As depicted in the figure, the obtained accuracy rate was 99.68%, 99.87%, 99.29%, and 99.64% for training, validation, test, and all data sets respectively.

## Discussion

BPH is the term used to describe the non-cancerous prostate enlargement which is frequently seen in elderly men. Almost all men start to experience significant prostate gland enlargement around the age of 40. Men in their 30 s and 40 s have a 10% prevalence of BPH, men in their 50 s and 60 s have fifty to sixty percent prevalence, and men in their 70 s and 80 s have 80% to 90% prevalence. Most men will undoubtedly experience some BPH symptoms if they live long enough^37^. Evidence-based Studies on the pathophysiology of BPH have demonstrated that inflammation has a role in the spread of histologic BPH^38,39^. It has been more difficult to figure out if the inflammation originated as a normal biologic result of ageing or whether it genuinely contributes in prostatic enlargement and the onset of LUTS^40^. Theoretically, antigenic stimuli may induce the prostate to establish a persistent inflammatory response that results in tissue regrowth and stromal expansion, leading to an autoimmune component to BPH^7,41^. The third factor in the pathogenesis of BPH can be regarded as chronic inflammation, which works in conjunction with androgen receptor signaling to cause the tissue remodeling that is typical of the disease's latter stages. The prostatic inflammation seen in BPH may result in the production of cytokines from inflammatory cells and a relative hypoxic situation brought on by the proliferating cells' increased oxygen demands, which could lead to tissue damage^42^.

Due to their advantages of simplicity and affordability, NLR, PLR, and NR have received a lot of attention as the most often utilized systemic inflammatory indicators. In particular, NLR might be used as a biomarker to determine the true nature of the mass; high NLR also suggested poor prognoses for malignancy in a number of tumor types^43^. However, recent research suggested that SII is a more potent method for predicting the development or spread of malignancies than NLR or NR^44,45^. In the proposed study, 40 individuals with symptomatic benign enlargement of the prostate had their NLR, PLR, and SII values assessed. NLR, PLR, and SII values were found to be effective predictors for disease severity, responsiveness to medical treatment, occurrence of urinary retention, and necessity for invasive intervention, according to the findings. They displayed 100% sensitivity and specificity to the disease, matching IPSS. As a result, they might be considered as a supplemental element when deciding on a treatment strategy. There is currently a lack of articles examining NLR, PLR, and SII in BPH patients. However, the predictive value of SII in gastrointestinal and urologic cancer patients was examined in some literature^14,46^. Moreover, Hu et al.^47^ reported that SII can be utilized as a predictive marker in hepatocellular carcinoma following curative resection.

Other studies have been also conducted to investigate the predictive potential of SII in a variety of disorders other than cancer (i.e., the pathophysiology of which includes chronic inflammation). After coronary intervention, complications like heart failure, cardiac death, non-fatal myocardial infarction, and non-fatal stroke were found to be more common with high SII values, according to a study by Yang et al.^48^. The authors came to the conclusion that SII might be a simple and effective indicator to identify high-risk patients before coronary intervention. A Number of studies has already examined the relationship between the NLR ratio and both cardiovascular and cancerous disorders^49,50^. Subsequently, it was reported that NLR was significant for several diseases' diagnostic and prognostic criteria^17,51,52^.

Damage to the prostatic tissue brought on by inflammation indicates a protracted process of wound repair that triggers hyperproliferative programs and leads to BPH nodules^53^. Prostatic enlargement may be caused directly by inflammatory activities that stimulate prostate growth or indirectly by inflammatory processes that reduce prostatic apoptosis. It was believed that histologic inflammation might be a sign of BPH progression and the necessity for invasive treatment^54^. BPH is considered to be an immune-mediated inflammatory disorder, and inflammation may directly influence prostate growth^8,55^. Based on previous clinical investigations, prostatic inflammations and lower urinary tract symptoms (LUTS) may be associated with BPH^4,7^.

In our proposed results, the IPSS score and accompanying NLR, PLR, and SII values were compared. In a group of 23 individuals with severe IPSS, high NLR values make up 70% of the group, high SII values make up 65%, and high PLR values make up 60%. On the other hand, as shown in Fig. 3, of the total 15 patients with moderate IPSS, 47% had high NLR values, 60% had high SII values, and 53% had high PLR values. Additionally, with increasing prostate size above cutoff value, 69% experienced high SII value and 61% of them showed high NLR while 58% exhibited high PLR As shown in Fig. 5a. Moreover, 53% of patients with PSA above the cutoff value showed high SII value and high NLR while only 50% showed high PLR as presented in Fig. 5b.

Although the occurrence of inflammatory infiltrates in human prostates is widely documented, the cause of inflammation in the prostate is still up for debate and is most likely to be complex. Inflammation in the prostate dramatically increases with age and prostate volume^37,56^. The advancement of symptoms, the possibility of urine retention, and the necessity for surgery are all associated with prostate inflammation^57^. In comparison to men treated for benign prostatic obstruction, patients sent to urology clinics with AUR were more likely to exhibit evidence of inflammation in prostatic specimens^58,59^. According to our ROC curves’ analysis, there was an equivalent relationship between SII, NLR, PLR, and BPH disease that was statistically significant to IPSS. The obtained ROC curve parameters utilizing SII, NLR, and PLR as indicators of BPH disease were the same as those that were obtained when IPSS was used to identify the disease. AUC was 1, while accuracy, sensitivity, and specificity were all 100% (Fig. 3 and Table 1). Therefore, we suggest that biomarkers based on inflammation can be utilized as a predictive biomarker to diagnose BPH, determine the course of treatment, the progression of the disease, the response to medication, and the need for invasive intervention. In this study, patients with high and moderate IPSS had significantly higher SII values, NLRs, and PLRs (Fig. 4). In cases where the NLR value was higher than the predetermined cut-off value, it was observed that there was a greater chance of refractory urine retention and the necessity for invasive intervention. Accordingly, one or more indicators like SII, NLR, or PLR may be taken into account before recommending medical care.

Although the routes of prostatic inflammation are not fully understood, mounting evidence points to the possibility that inflammatory processes affecting both the prostate and the bladder may be crucial to the initiation and maintenance of prostate growth and LUTS. Immunologic processes and inflammation are either implicated in the pathophysiology of all prostatic disorders or are considered as potential initiators of disease progression. The stimulation of stromal and epithelial cell proliferation that is sustained by autoimmune mechanisms may be caused by T-cell activation in inflammatory infiltrates. BPH nodules grow as a result of tissue injury and the ongoing, chronic process of recurrent wound repair brought on by inflammation. The systemic SII, NLR, and PLR, which are based on peripheral platelet, neutrophil, and lymphocyte counts, have been shown to be promising prognostic predictors in figuring out the severity of symptoms, how well it responds to medical treatment, the frequency of urinary retention, and the need for invasive intervention.

## Conclusions

The study showed that SII, NLR, and PLR are all distinct risk factors for the diagnosis of BPH and the progression of IPSS. The SII, NLR, and PLR can be used to anticipate the inflammatory condition when treating BPH patients. We came to the conclusion that SII, NLR, and PLR are significant inflammatory biomarkers for detecting BPH and directing treatment choices. Variations in SII, NLR, and PLR may be able to predict clinical outcome and response, offering a relatively straightforward way to track therapy effectiveness. Regular laboratory testing can quickly and affordably determine it, giving it wide availability. The relatively small sample size, retrospective nature, and use of a single blood sample are among the limitations of this study. Therefore, prospective studies with large samples and the collection of different blood samples from each patient are required to corroborate our findings.

### Supplementary Information

Supplementary Information 1.

Supplementary Information 2.

## Data Availability

The datasets used and/or analyzed during the current study are available from the corresponding author on reasonable request.
