# Supplementary material for: Diagnostic efficacy of systemic immune-inflammation biomarkers in benign prostatic hyperplasia using receiver operating characteristic and artificial neural network
Source: Sci Rep. 2023 Sep 8;13:14801. doi: 10.1038/s41598-023-41781-3 (PMC10491602; doi:10.1038/s41598-023-41781-3)
Supplement: Supplementary file 1 — Supplementary Information 1. [file 41598_2023_41781_MOESM1_ESM.pdf]

```
EXAMINE VARIABLES=Size_Non_BPH PSA_Non_BPH PLR_Non_BPH NLR_Non_BPH SII_Non_BPH
 IPSS_BPH Size_BPH
    PSA_BPH PLR_BPH NLR_BPH S11_BPH
  /PLOT BOXPLOT STEMLEAF HISTOGRAM NPPLOT
  /COMPARE VARIABLES
  /STATISTICS DESCRIPTIVES
  /CINTERVAL 95
  /MISSING LISTWISE
  /NOTOTAL.
```

Explore

Notes

Output Created		31-JUL-2023 14:34:52
Comments		
Input	Data	C:\Users\Lenovo\Desktop\Untitled1.sav
	Active Dataset	DataSet1
	Filter	<none>
	Weight	<none>
	Split File	<none>
	N of Rows in Working Data File	80
Missing Value Handling	Definition of Missing	User-defined missing values for dependent variables are treated as missing.
	Cases Used	Statistics are based on cases with no missing values for any dependent variable or factor used.

Notes

Syntax		EXAMINE VARIABLES=Size_Non_BPH PSA_Non_BPH PLR_Non_BPH NLR_Non_BPH SII_Non_BPH IPSS_BPH Size_BPH PSA_BPH PLR_BPH NLR_BPH S11_BPH /PLOT BOXPLOT STEMLEAF HISTOGRAM NPPLOT /COMPARE VARIABLES /STATISTICS DESCRIPTIVES /CINTERVAL 95 /MISSING LISTWISE /NOTOTAL.
Resources	Processor Time	00:00:04.70
	Elapsed Time	00:00:04.37

Case Processing Summary

	Cases					
	Valid		Missing		Total	
	N	Percent	N	Percent	N	Percent
Size_Non_BPH	40	50.0%	40	50.0%	80	100.0%
PSA_Non_BPH	40	50.0%	40	50.0%	80	100.0%
PLR_Non_BPH	40	50.0%	40	50.0%	80	100.0%
NLR_Non_BPH	40	50.0%	40	50.0%	80	100.0%
SII_Non_BPH	40	50.0%	40	50.0%	80	100.0%
IPSS_BPH	40	50.0%	40	50.0%	80	100.0%
Size_BPH	40	50.0%	40	50.0%	80	100.0%
PSA_BPH	40	50.0%	40	50.0%	80	100.0%
PLR_BPH	40	50.0%	40	50.0%	80	100.0%
NLR_BPH	40	50.0%	40	50.0%	80	100.0%
S11_BPH	40	50.0%	40	50.0%	80	100.0%

Descriptives

			Statistic	Std. Error
Size_Non_BPH	Mean		28.1000	.67729
	95% Confidence Interval for Mean	Lower Bound	26.7301	
		Upper Bound	29.4699	
	5% Trimmed Mean		28.0556	
	Median		28.5000	
	Variance		18.349	
	Std. Deviation		4.28354	
	Minimum		22.00	
	Maximum		35.00	
	Range		13.00	
	Interquartile Range		5.00	
	Skewness		.076	.374
	Kurtosis		-.976	.733
PSA_Non_BPH	Mean		2.3000	.11255
	95% Confidence Interval for Mean	Lower Bound	2.0724	
		Upper Bound	2.5276	
	5% Trimmed Mean		2.3278	
	Median		2.2500	
	Variance		.507	
	Std. Deviation		.71181	
	Minimum		.90	
	Maximum		3.20	
	Range		2.30	
	Interquartile Range		1.20	
	Skewness		-.449	.374
	Kurtosis		-.767	.733
PLR_Non_BPH	Mean		.1329	.01335
	95% Confidence Interval for Mean	Lower Bound	.1058	
		Upper Bound	.1599	
	5% Trimmed Mean		.1252	
	Median		.1007	
	Variance		.007	
	Std. Deviation		.08446	
	Minimum		.05	
	Maximum		.40	

Descriptives

			Statistic	Std. Error
	Range		.36	
	Interquartile Range		.09	
	Skewness		1.479	.374
	Kurtosis		1.735	.733
NLR_Non_BPH	Mean		2.3058	.24418
	95% Confidence Interval for Mean	Lower Bound	1.8119	
		Upper Bound	2.7997	
	5% Trimmed Mean		2.2007	
	Median		1.6807	
	Variance		2.385	
	Std. Deviation		1.54434	
	Minimum		.67	
	Maximum		5.94	
	Range		5.28	
	Interquartile Range		1.75	
	Skewness		1.219	.374
	Kurtosis		.206	.733
SII_Non_BPH	Mean		637.2571	88.89313
	95% Confidence Interval for Mean	Lower Bound	457.4538	
		Upper Bound	817.0605	
	5% Trimmed Mean		576.8220	
	Median		394.1345	
	Variance		316079.567	
	Std. Deviation		562.20954	
	Minimum		139.30	
	Maximum		2537.83	
	Range		2398.53	
	Interquartile Range		585.96	
	Skewness		1.741	.374
	Kurtosis		2.660	.733
IPSS_BPH	Mean		20.6250	1.25560
	95% Confidence Interval for Mean	Lower Bound	18.0853	
		Upper Bound	23.1647	
	5% Trimmed Mean		20.5556	
	Median		20.0000	

Descriptives

			Statistic	Std. Error
	Variance		63.061	
	Std. Deviation		7.94109	
	Minimum		5.00	
	Maximum		35.00	
	Range		30.00	
	Interquartile Range		10.00	
	Skewness		.229	.374
	Kurtosis		-.704	.733
Size_BPH	Mean		92.9500	7.80072
	95% Confidence Interval for Mean	Lower Bound	77.1716	
		Upper Bound	108.7284	
	5% Trimmed Mean		88.7500	
	Median		82.5000	
	Variance		2434.049	
	Std. Deviation		49.33608	
	Minimum		25.00	
	Maximum		285.00	
	Range		260.00	
	Interquartile Range		38.25	
	Skewness		1.845	.374
	Kurtosis		5.050	.733
PSA_BPH	Mean		6.5822	1.09394
	95% Confidence Interval for Mean	Lower Bound	4.3695	
		Upper Bound	8.7950	
	5% Trimmed Mean		5.5225	
	Median		4.3500	
	Variance		47.868	
	Std. Deviation		6.91870	
	Minimum		.80	
	Maximum		41.50	
	Range		40.70	
	Interquartile Range		5.18	
	Skewness		3.691	.374
	Kurtosis		16.914	.733
PLR_BPH	Mean		120.8840	13.83808

Descriptives

			Statistic	Std. Error
	95% Confidence Interval for Mean	Lower Bound	92.8939	
		Upper Bound	148.8742	
	5% Trimmed Mean		108.4390	
	Median		103.1034	
	Variance		7659.695	
	Std. Deviation		87.51968	
	Minimum		45.01	
	Maximum		542.86	
	Range		497.85	
	Interquartile Range		78.74	
	Skewness		3.234	.374
	Kurtosis		13.629	.733
NLR_BPH	Mean		120.8840	13.83808
	95% Confidence Interval for Mean	Lower Bound	92.8939	
		Upper Bound	148.8742	
	5% Trimmed Mean		108.4390	
	Median		103.1034	
	Variance		7659.695	
	Std. Deviation		87.51968	
	Minimum		45.01	
	Maximum		542.86	
	Range		497.85	
	Interquartile Range		78.74	
	Skewness		3.234	.374
	Kurtosis		13.629	.733
S11_BPH	Mean		607725.9560	81263.12350
	95% Confidence Interval for Mean	Lower Bound	443355.7740	
		Upper Bound	772096.1381	
	5% Trimmed Mean		536331.0384	
	Median		453731.1912	
	Variance		2.641E+11	
	Std. Deviation		513953.1201	
	Minimum		168341.46	
	Maximum		2.61E+6	
	Range		2437372.82	

Descriptives

		Statistic	Std. Error
	Interquartile Range	451294.38	
	Skewness	2.288	.374
	Kurtosis	5.998	.733

Tests of Normality

	Kolmogorov-Smirnov[a]			Shapiro-Wilk		
	Statistic	df	Sig.	Statistic	df	Sig.
Size_Non_BPH	.129	40	.093	.914	40	.005
PSA_Non_BPH	.200	40	.000	.889	40	.001
PLR_Non_BPH	.204	40	.000	.826	40	.000
NLR_Non_BPH	.254	40	.000	.810	40	.000
SII_Non_BPH	.226	40	.000	.776	40	.000
IPSS_BPH	.161	40	.011	.943	40	.044
Size_BPH	.172	40	.004	.846	40	.000
PSA_BPH	.221	40	.000	.616	40	.000
PLR_BPH	.223	40	.000	.673	40	.000
NLR_BPH	.223	40	.000	.673	40	.000
S11_BPH	.219	40	.000	.747	40	.000

a. Lilliefors Significance Correction

Histograms

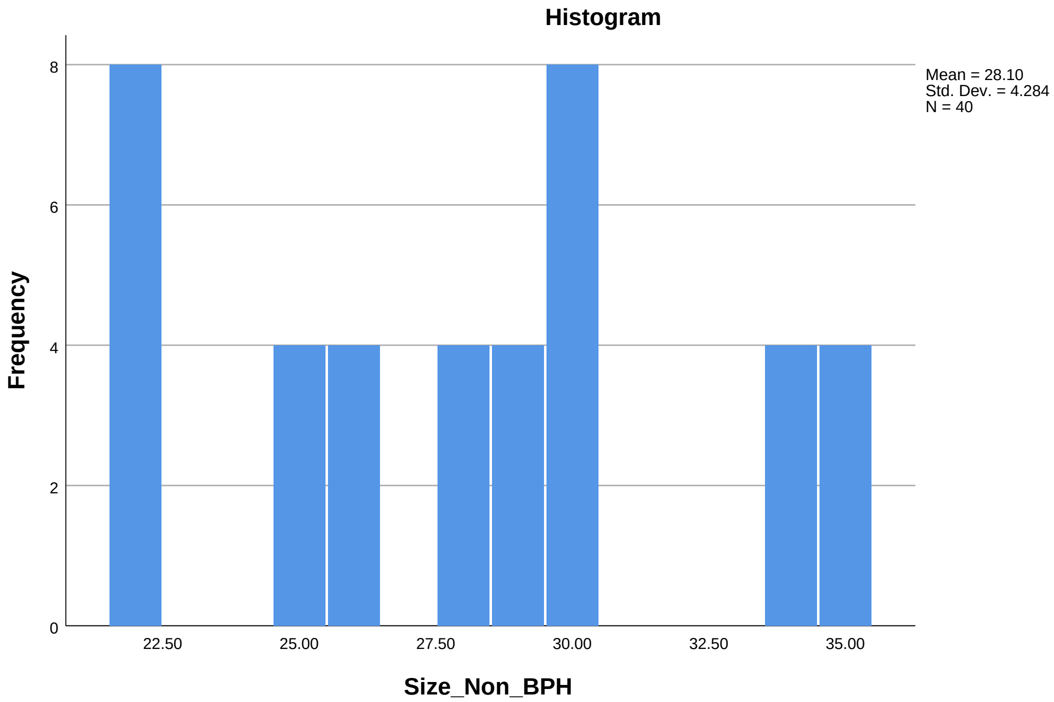
Histogram
Mean = 28.10
Std. Dev. = 4.284
N = 40
Frequency
0
2
4
6
8
22.50
25.00
27.50
30.00
32.50
35.00
Size_Non_BPH

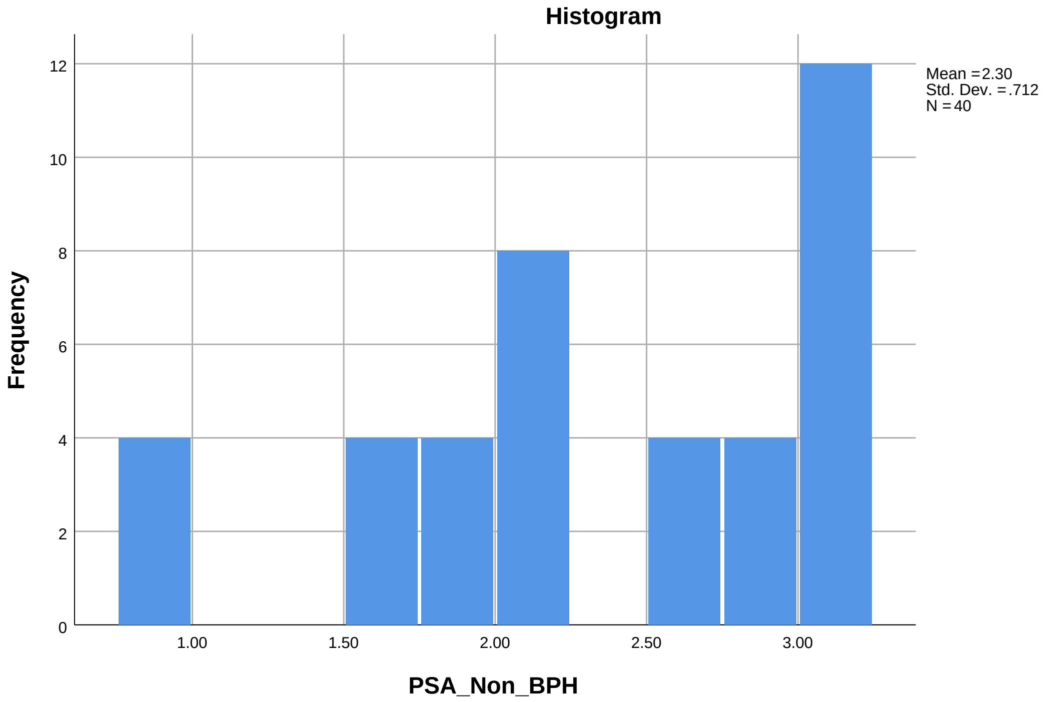
Histogram
Mean =2.30
Std. Dev. =.712
N =40
Frequency
0
2
4
6
8
10
12
1.00
1.50
2.00
2.50
3.00
PSA_Non_BPH

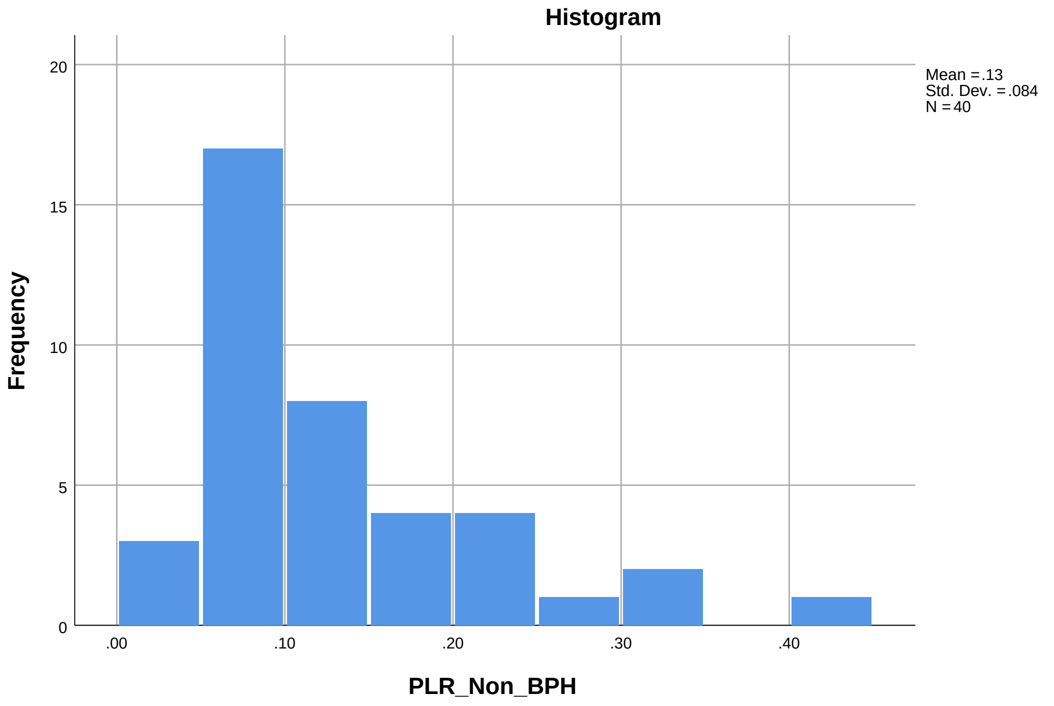
Histogram
Mean =.13
Std. Dev. =.084
N =40
Frequency
20
15
10
5
0
.00
.10
.20
.30
.40
PLR_Non_BPH

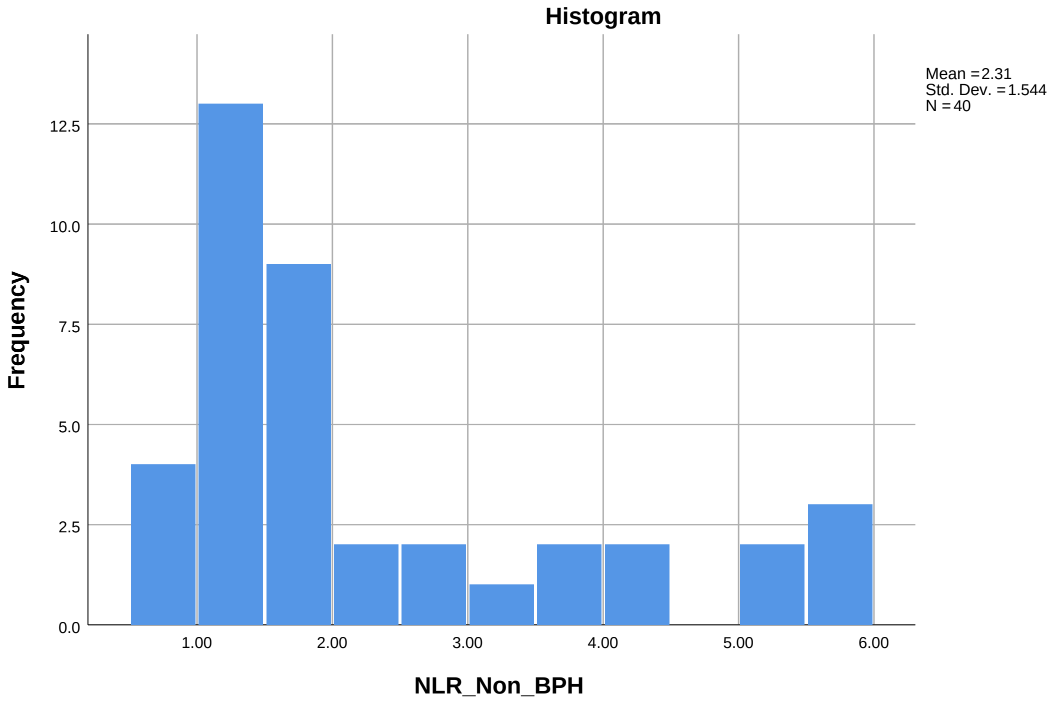
Histogram
Mean =2.31
Std. Dev. =1.544
N =40
Frequency
12.5
10.0
7.5
5.0
2.5
0.0
1.00
2.00
3.00
4.00
5.00
6.00
NLR_Non_BPH

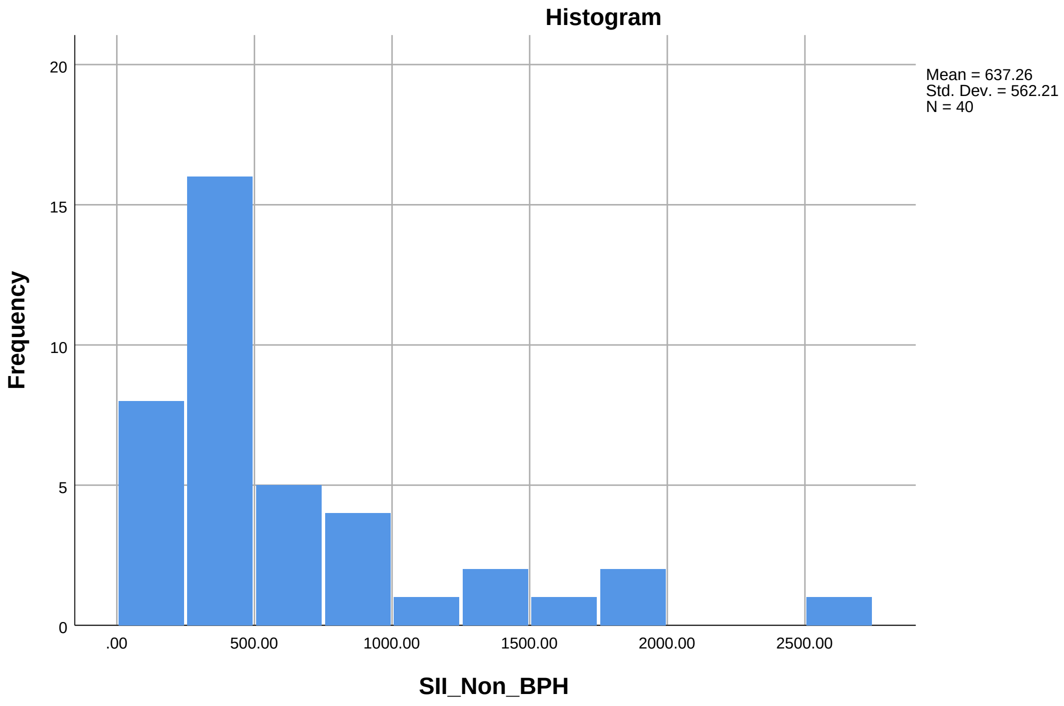
Histogram
Mean = 637.26
Std. Dev. = 562.21
N = 40
Frequency
20
15
10
5
0
.00
500.00
1000.00
1500.00
2000.00
2500.00
SII_Non_BPH

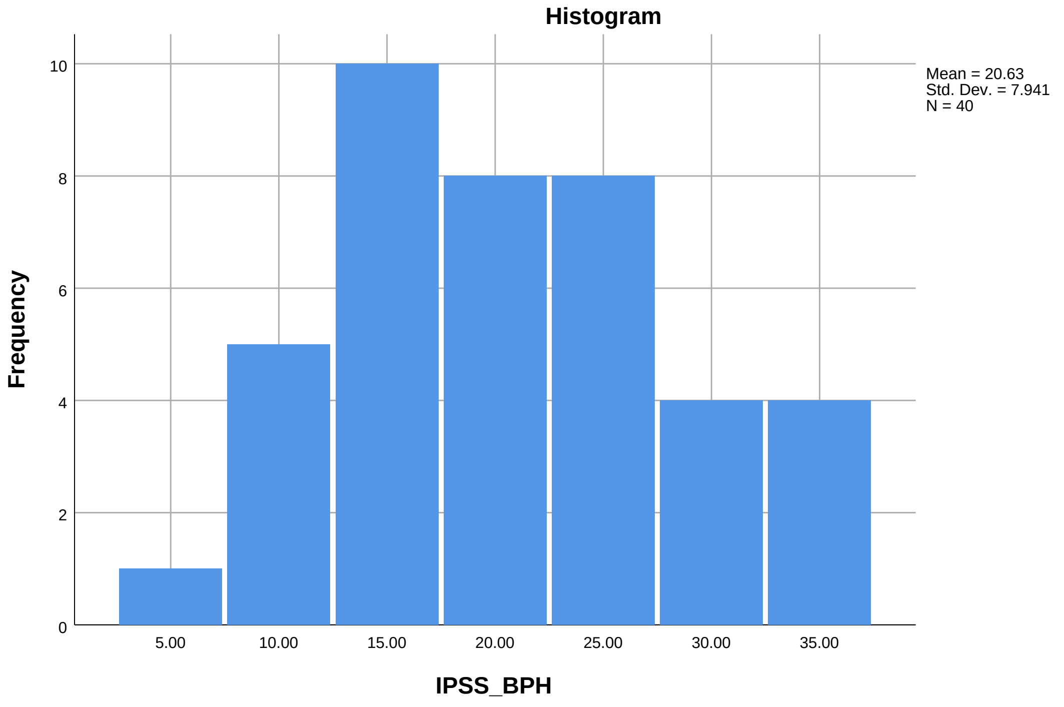
Histogram
Mean = 20.63
Std. Dev. = 7.941
N = 40
Frequency
10
8
6
4
2
0
5.00
10.00
15.00
20.00
25.00
30.00
35.00
IPSS_BPH

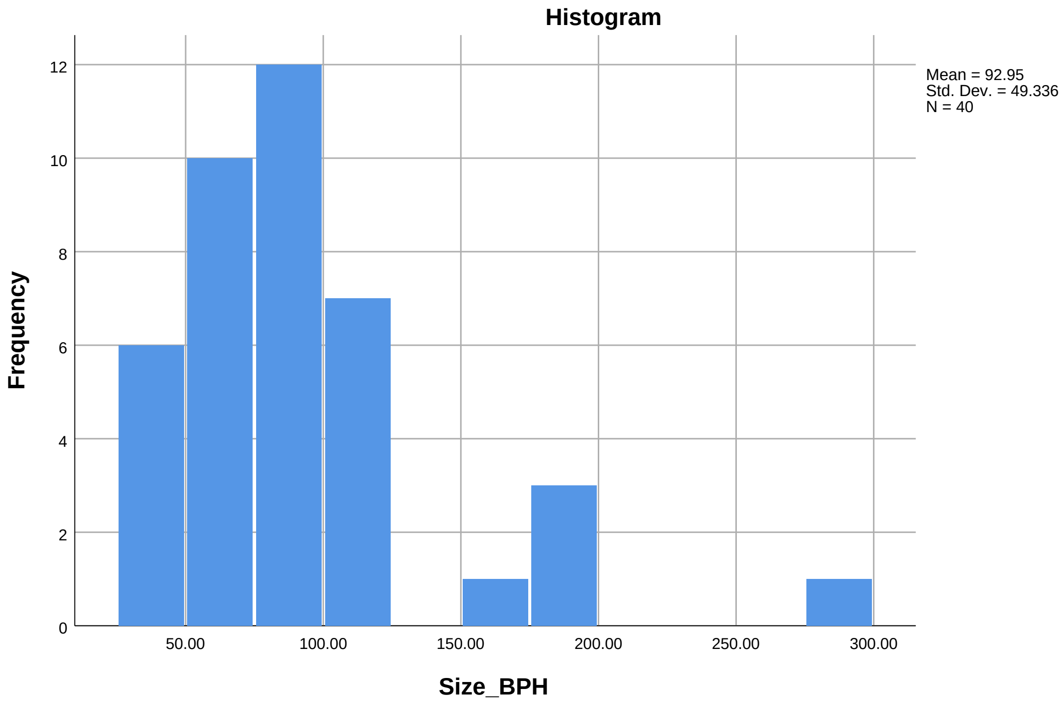
Histogram
Mean = 92.95
Std. Dev. = 49.336
N = 40
Frequency
0
2
4
6
8
10
12
50.00
100.00
150.00
200.00
250.00
300.00
Size_BPH

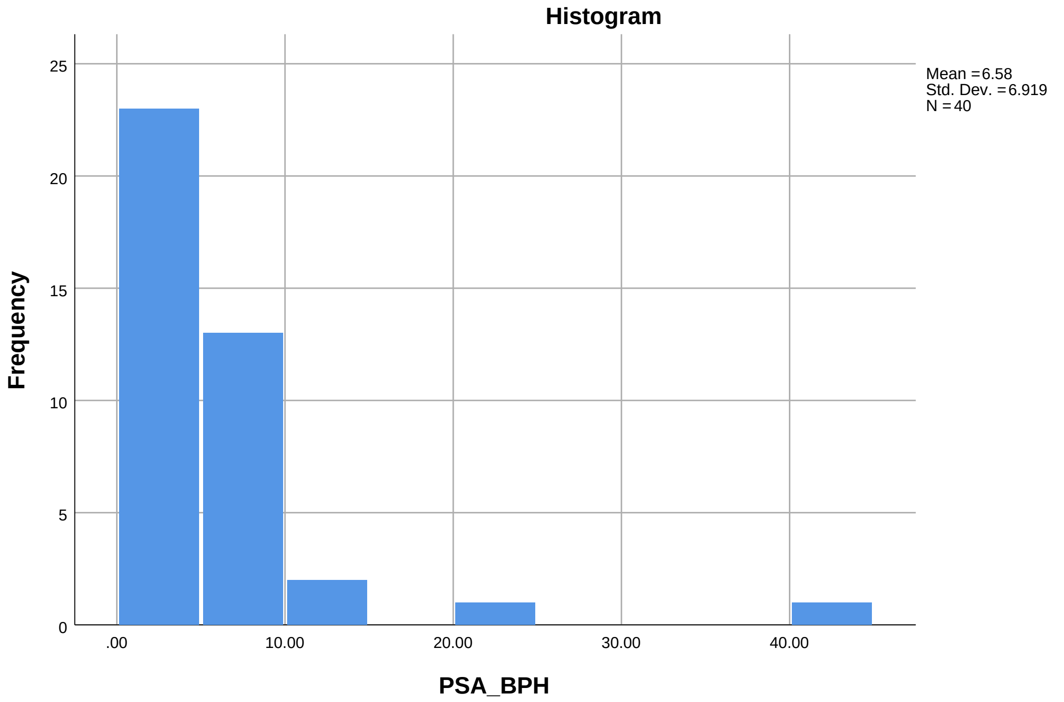
Histogram
Mean =6.58
Std. Dev. =6.919
N =40
Frequency
0
5
10
15
20
25
.00
10.00
20.00
30.00
40.00
PSA_BPH

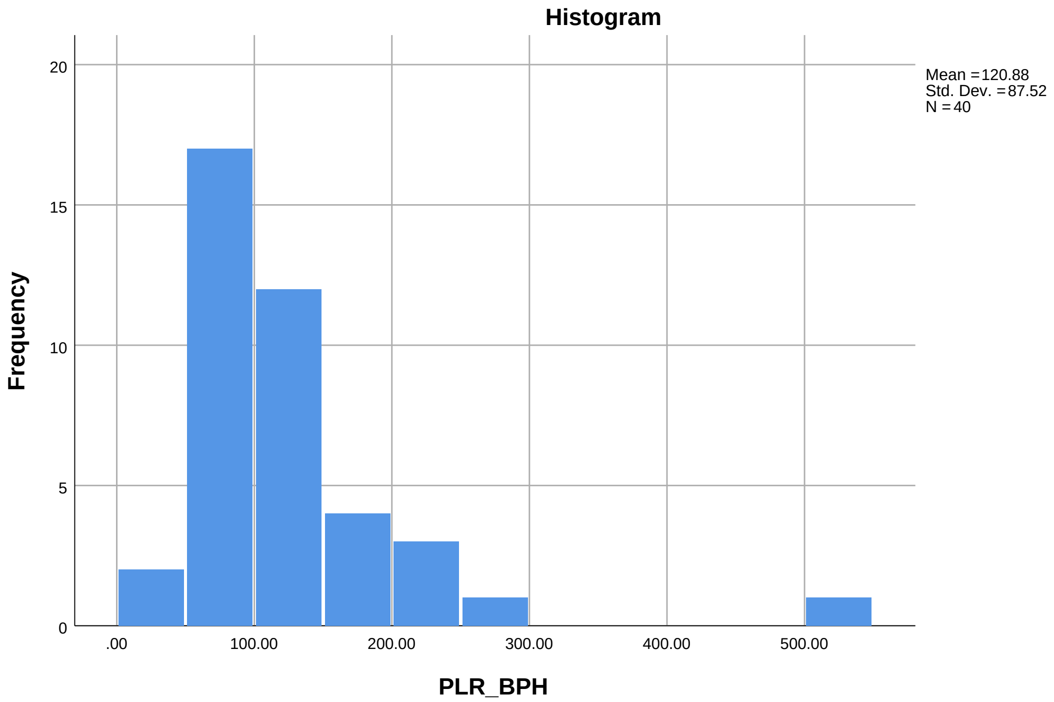
Histogram
Mean = 120.88
Std. Dev. = 87.52
N = 40
Frequency
PLR_BPH

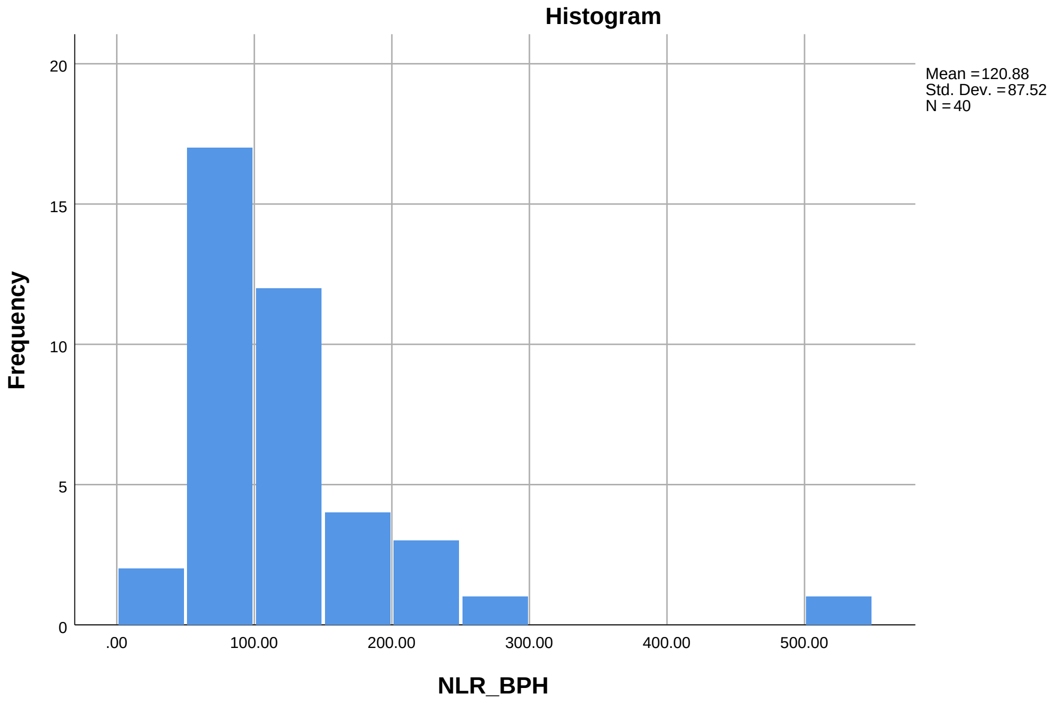
Histogram
Mean = 120.88
Std. Dev. = 87.52
N = 40
Frequency
NLR_BPH

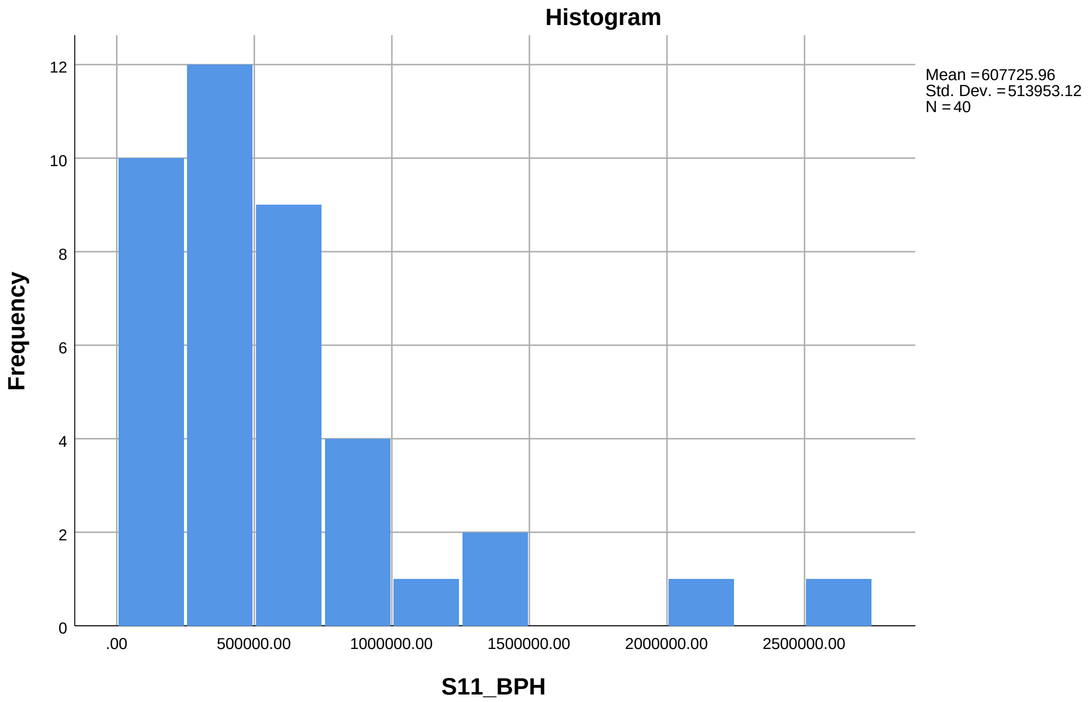

Stem-and-Leaf Plots

```
Size_Non_BPH Stem-and-Leaf Plot

 Frequency    Stem &  Leaf

      .00        2 .
     8.00        2 .  22222222
     4.00        2 .  5555
     4.00        2 .  6666
     8.00        2 .  88889999
     8.00        3 .  00000000
      .00        3 .
     8.00        3 .  44445555

 Stem width:     10.00
 Each leaf:        1 case(s)



PSA_Non_BPH Stem-and-Leaf Plot

 Frequency    Stem &  Leaf
```

```
     4.00        0 .  9999
      .00        1 .
     8.00        1 .  77778888
     8.00        2 .  00000000
     8.00        2 .  55559999
    12.00        3 .  000000002222

 Stem width:      1.00
 Each leaf:        1 case(s)
```

PLR_Non_BPH Stem-and-Leaf Plot

```
 Frequency    Stem &  Leaf

     3.00        0 .  444
    17.00        0 .  66677777777888999
     8.00        1 .  00012344
     4.00        1 .  5569
     4.00        2 .  1233
     4.00 Extremes    (>=.29)

 Stem width:       .10
 Each leaf:        1 case(s)
```

NLR_Non_BPH Stem-and-Leaf Plot

```
 Frequency    Stem &  Leaf

     4.00        0 .  6789
    22.00        1 .  0001233333444556677789
     4.00        2 .  1277
     3.00        3 .  159
     2.00        4 .  34
     2.00        5 .  13
     3.00 Extremes    (>=5.5)

 Stem width:      1.00
```

```
 Each leaf:        1 case(s)
```

SII_Non_BPH Stem-and-Leaf Plot

```
 Frequency    Stem &  Leaf

     3.00        0 .  111
    17.00        0 .  22222222223333333
     7.00        0 .  4444555
     2.00        0 .  67
     4.00        0 .  8889
      .00        1 .
     2.00        1 .  22
     1.00        1 .  4
     1.00        1 .  6
     3.00 Extremes    (>=1767)

 Stem width:   1000.00
 Each leaf:        1 case(s)
```

IPSS_BPH Stem-and-Leaf Plot

```
 Frequency    Stem &  Leaf

      .00        0 .
     1.00        0 .  5
     5.00        1 .  00000
    10.00        1 .  5555555555
     8.00        2 .  00000000
     8.00        2 .  55555555
     4.00        3 .  0000
     4.00        3 .  5555

 Stem width:     10.00
 Each leaf:        1 case(s)
```

Size_BPH Stem-and-Leaf Plot

```
 Frequency    Stem &  Leaf

     2.00        0 .  22
     5.00        0 .  44445
    10.00        0 .  6667777777
    11.00        0 .  88888999999
     5.00        1 .  00001
     2.00        1 .  22
     1.00        1 .  5
     4.00 Extremes    (>=182)

 Stem width:    100.00
 Each leaf:        1 case(s)
```

PSA_BPH Stem-and-Leaf Plot

```
 Frequency    Stem &  Leaf

     5.00        0 .  00111
    10.00        0 .  2222333333
     9.00        0 .  444444445
     6.00        0 .  666677
     6.00        0 .  888999
     1.00        1 .  1
      .00        1 .
     1.00        1 .  4
     2.00 Extremes    (>=21)

 Stem width:     10.00
 Each leaf:        1 case(s)
```

PLR_BPH Stem-and-Leaf Plot

```
 Frequency    Stem &  Leaf

     2.00        0 .  44
    17.00        0 .  56666666677777899
```

```
   12.00        1 .  000000111244
    4.00        1 .  5666
    3.00        2 .  003
    2.00 Extremes    (>=296)

 Stem width:    100.00
 Each leaf:        1 case(s)
```

NLR_BPH Stem-and-Leaf Plot

```
 Frequency    Stem &  Leaf

    2.00        0 .  44
   17.00        0 .  56666666677777899
   12.00        1 .  000000111244
    4.00        1 .  5666
    3.00        2 .  003
    2.00 Extremes    (>=296)

 Stem width:    100.00
 Each leaf:        1 case(s)
```

S11_BPH Stem-and-Leaf Plot

```
 Frequency    Stem &  Leaf

    3.00        0 .  111
   14.00        0 .  22222222233333
    9.00        0 .  444445555
    6.00        0 .  666677
    3.00        0 .  899
    1.00        1 .  1
    4.00 Extremes    (>=1372800)

 Stem width:   1000000
 Each leaf:        1 case(s)
```

Normal Q-Q Plots

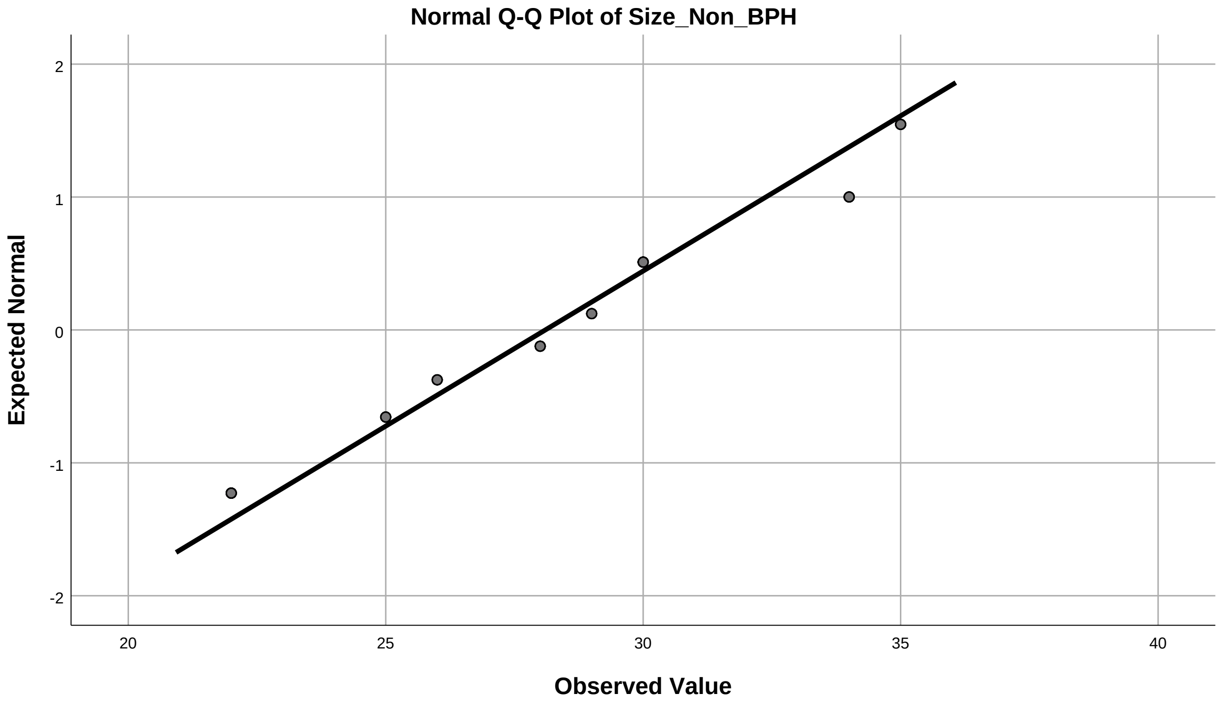

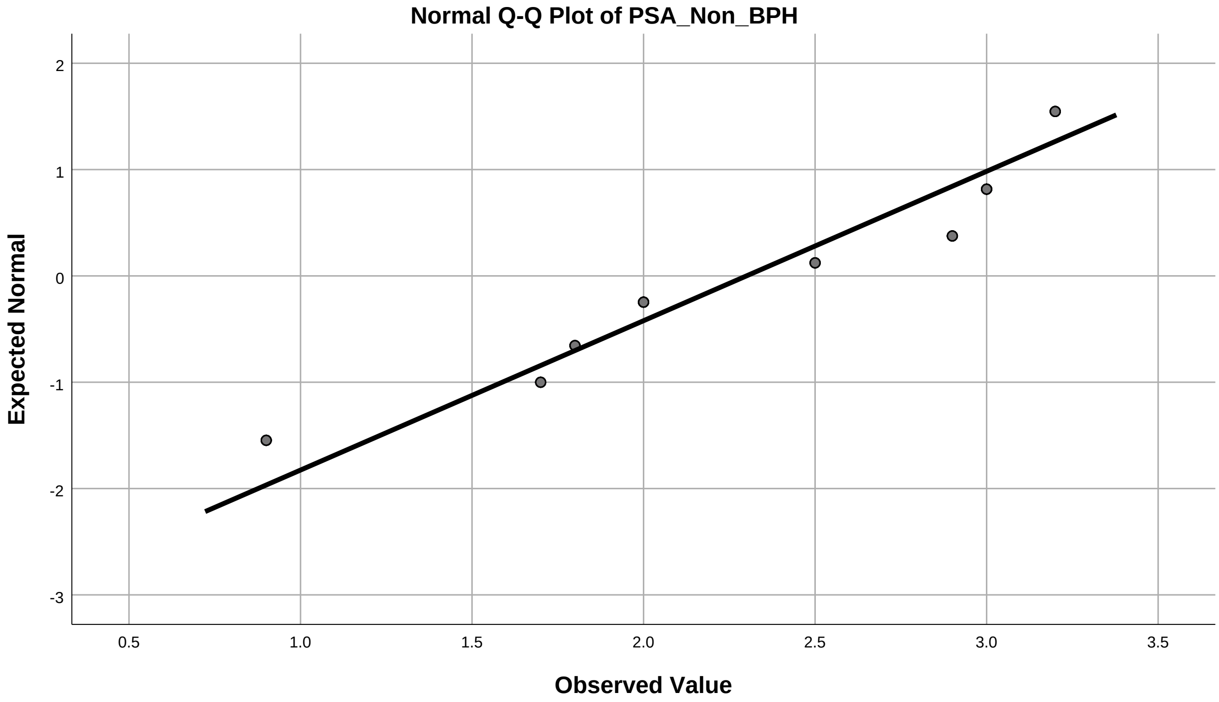

Normal Q-Q Plot of PLR_Non_BPH

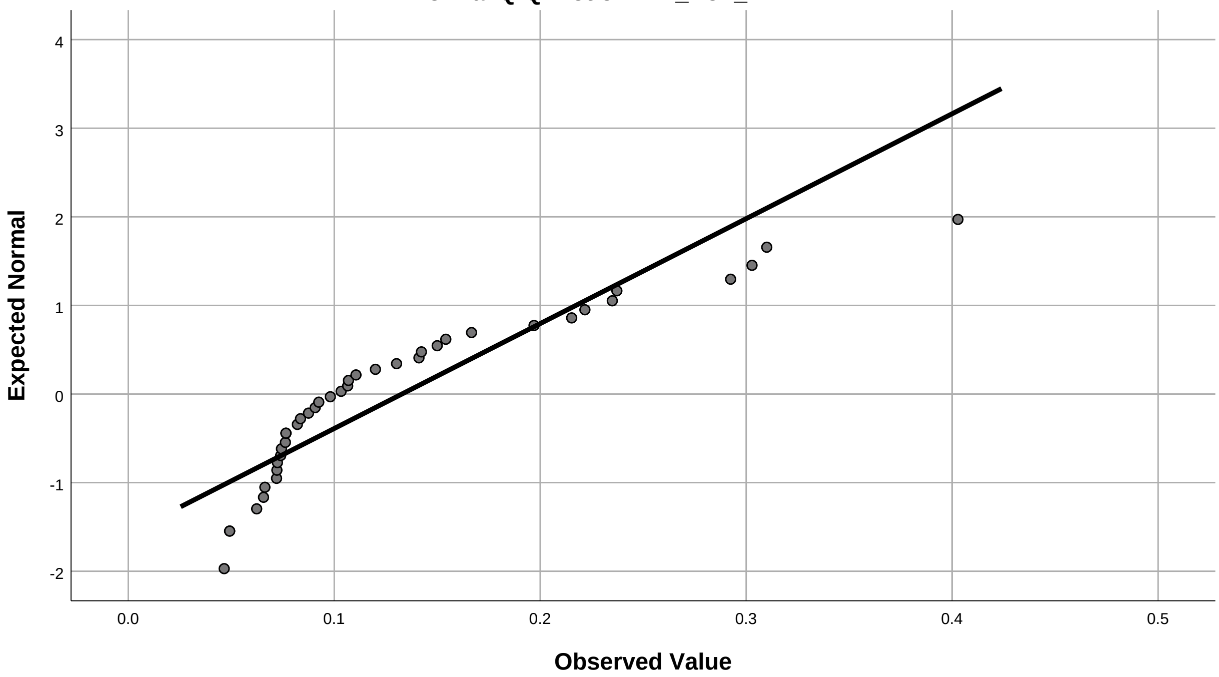

Normal Q-Q Plot of NLR_Non_BPH

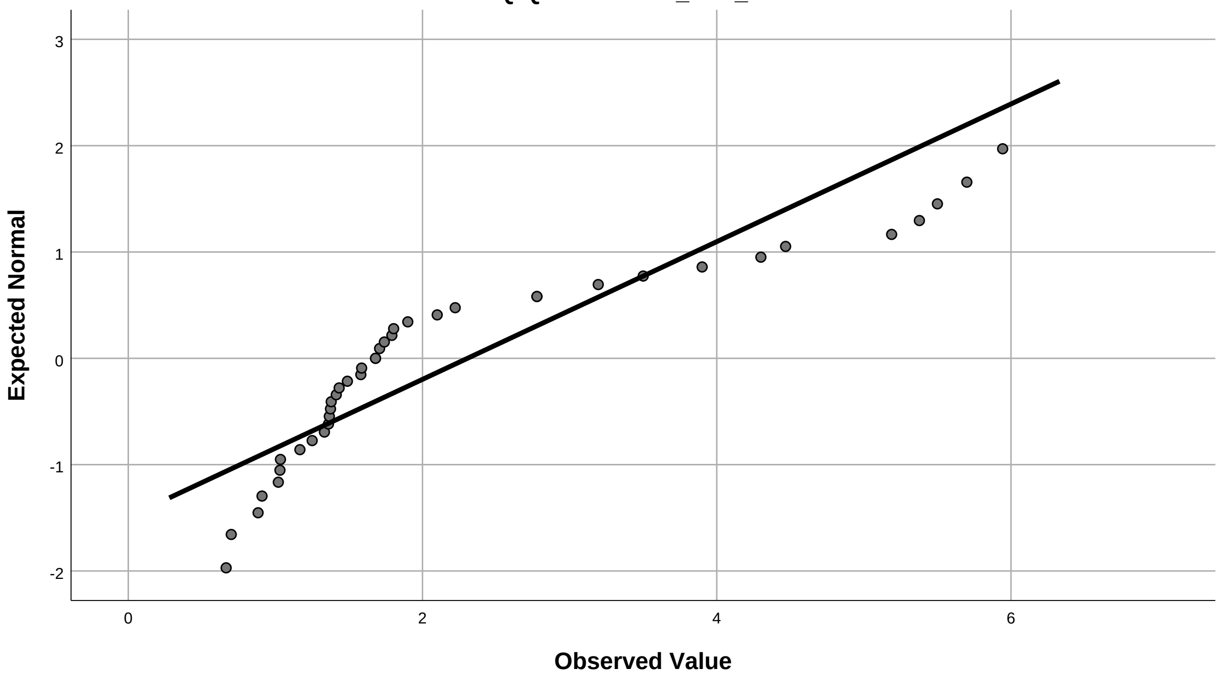

Normal Q-Q Plot of SII_Non_BPH

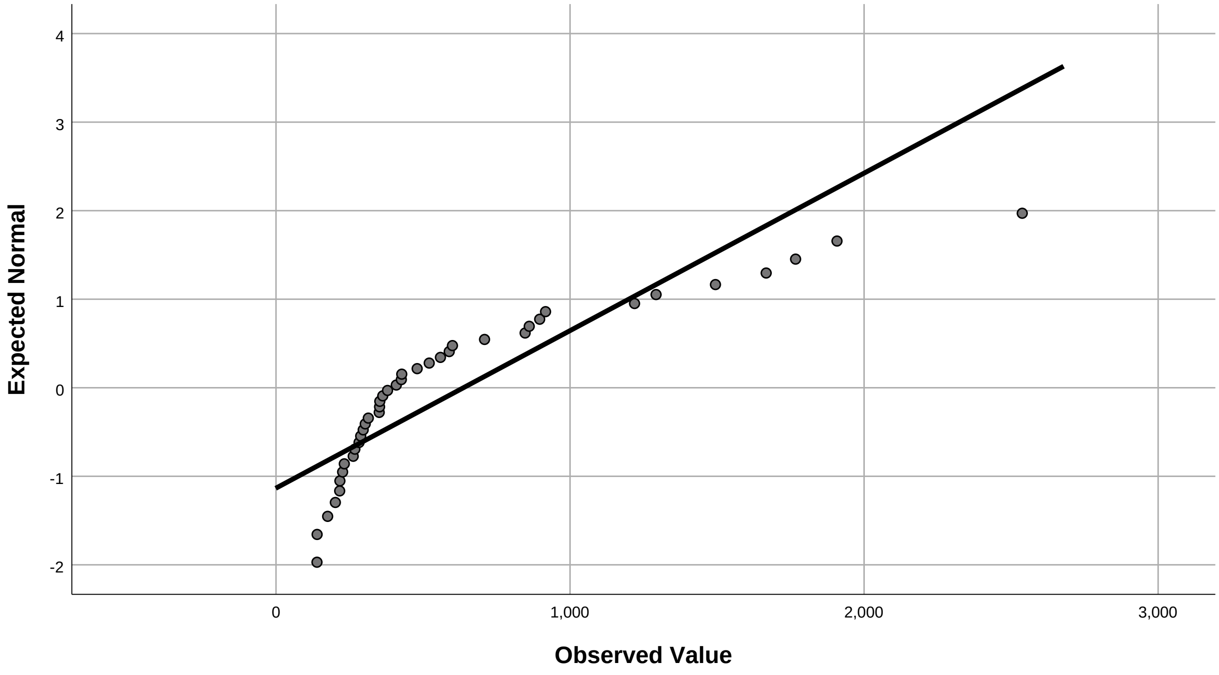

Normal Q-Q Plot of IPSS_BPH

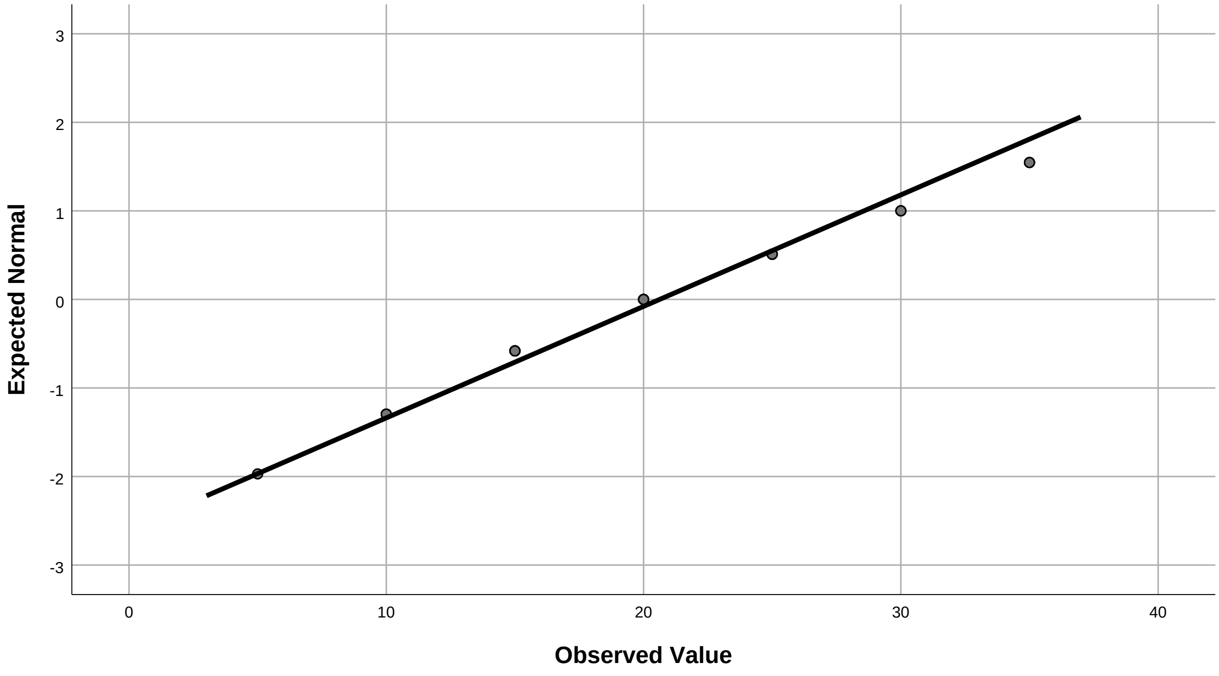

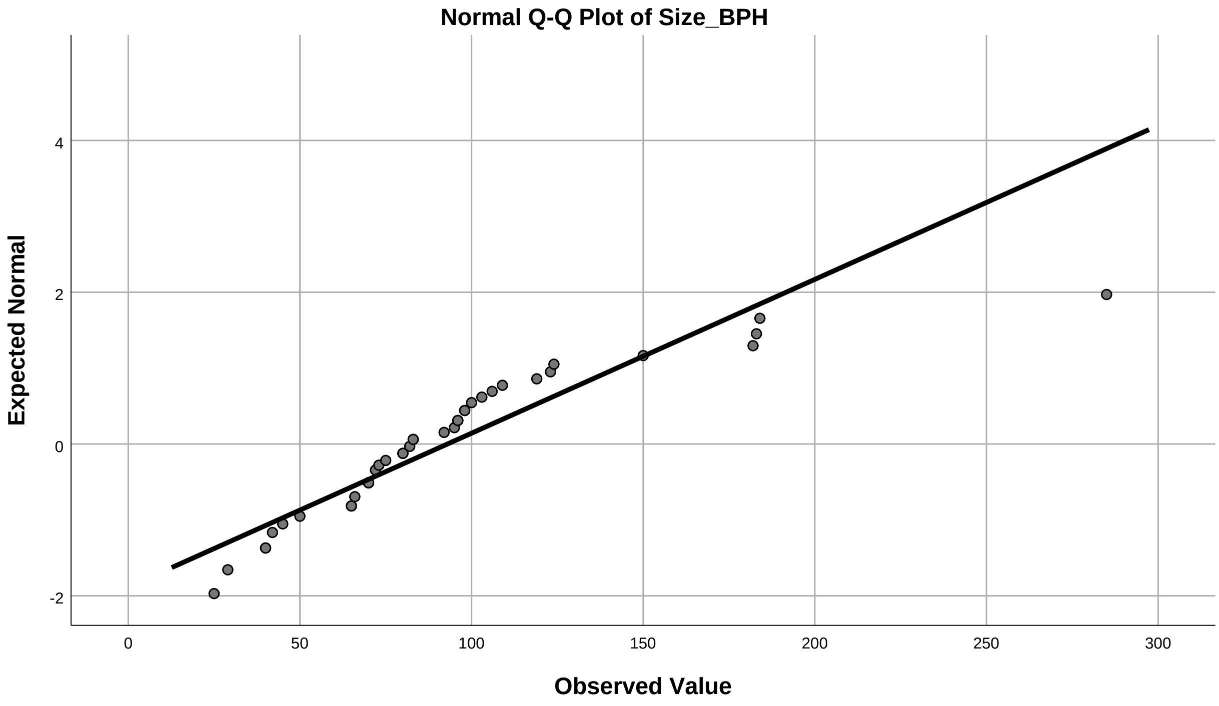
Normal Q-Q Plot of Size_BPH
Expected Normal
Observed Value
4
2
0
-2
0
50
100
150
200
250
300

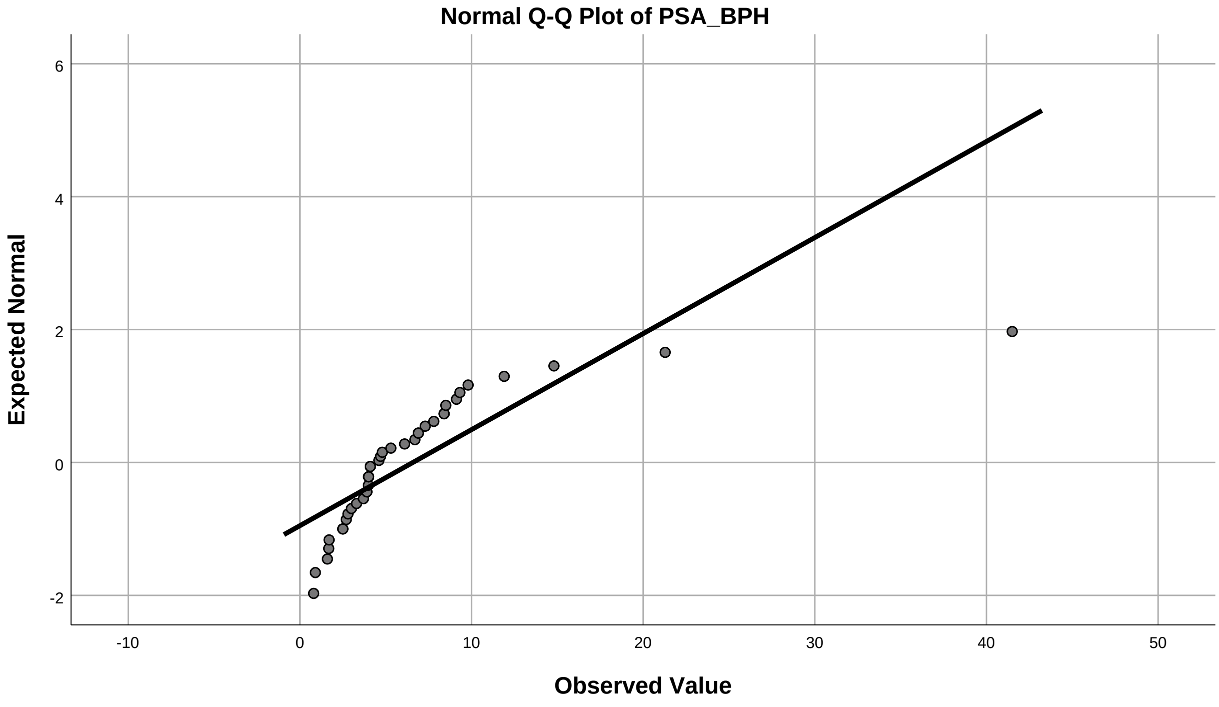
Normal Q-Q Plot of PSA_BPH
Expected Normal
Observed Value
6
4
2
0
-2
-10
0
10
20
30
40
50

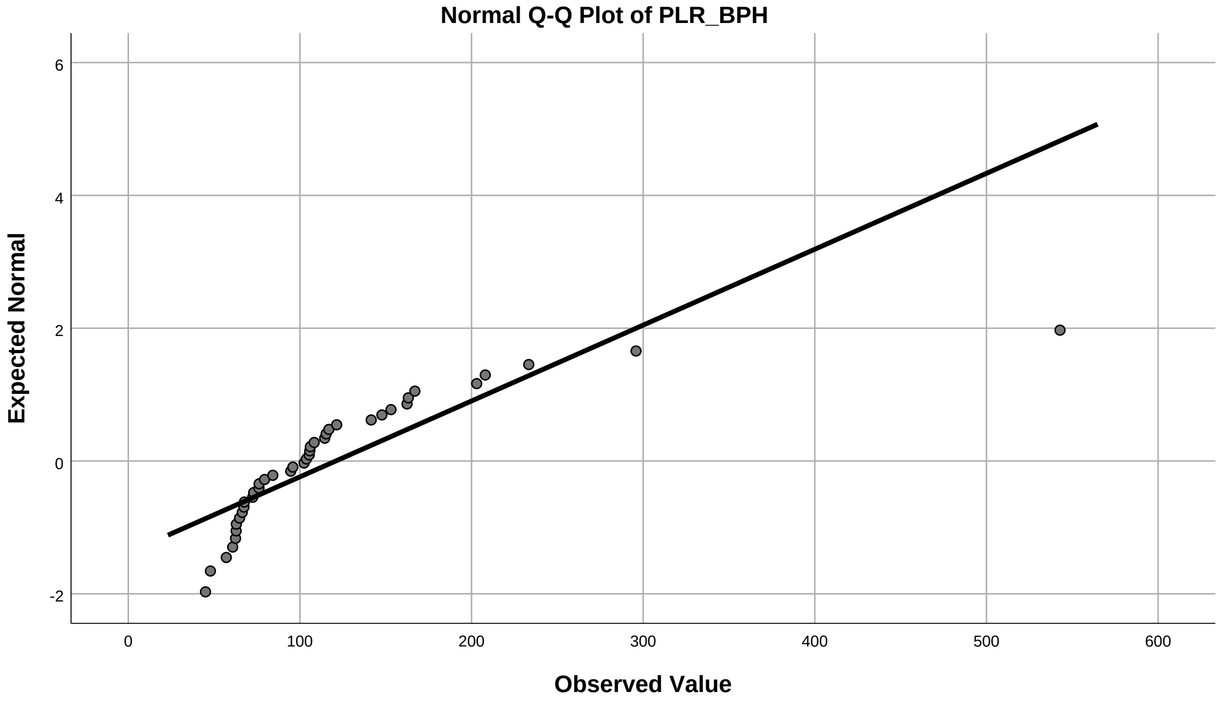
Normal Q-Q Plot of PLR_BPH
Expected Normal
Observed Value
6
4
2
0
-2
0
100
200
300
400
500
600

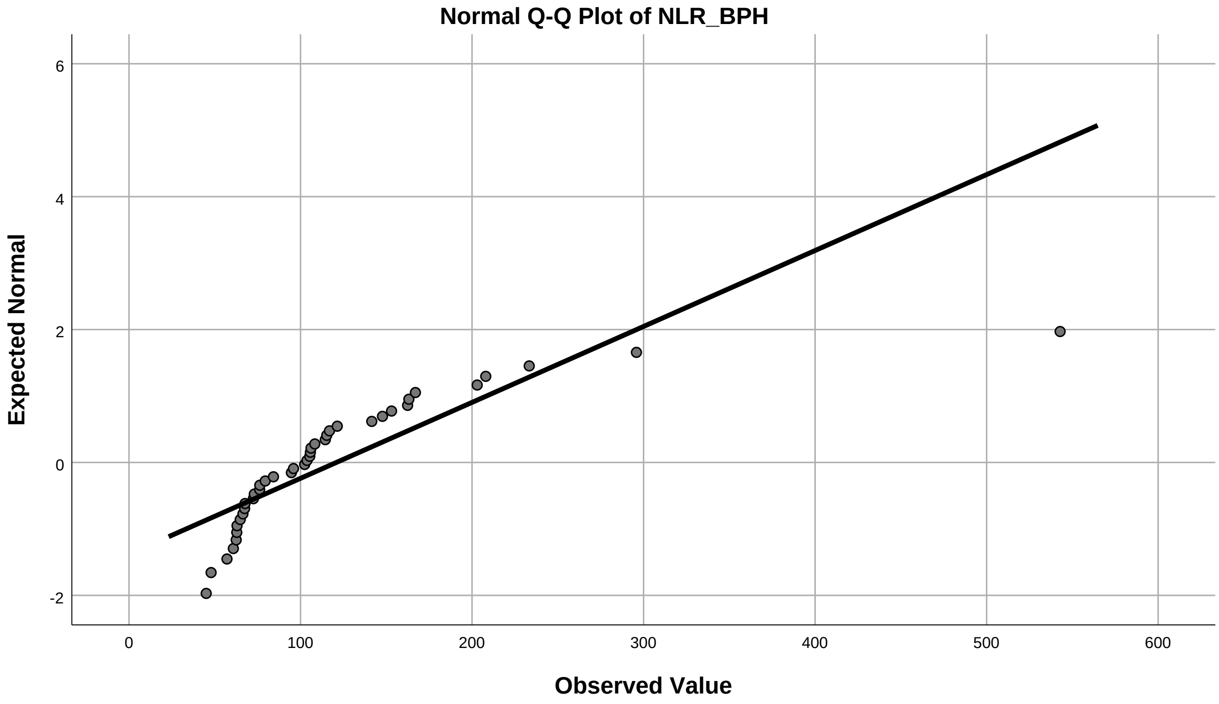
Normal Q-Q Plot of NLR_BPH
Expected Normal
Observed Value
6
4
2
0
-2
0
100
200
300
400
500
600

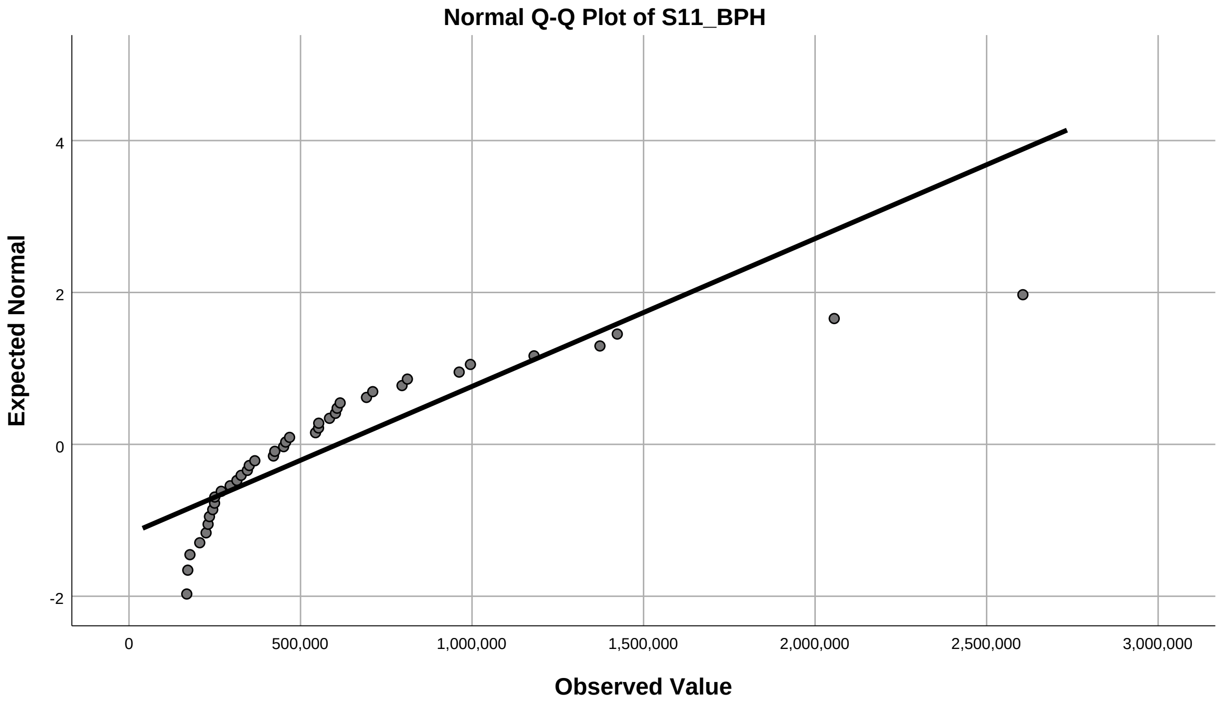

Detrended Normal Q-Q Plots

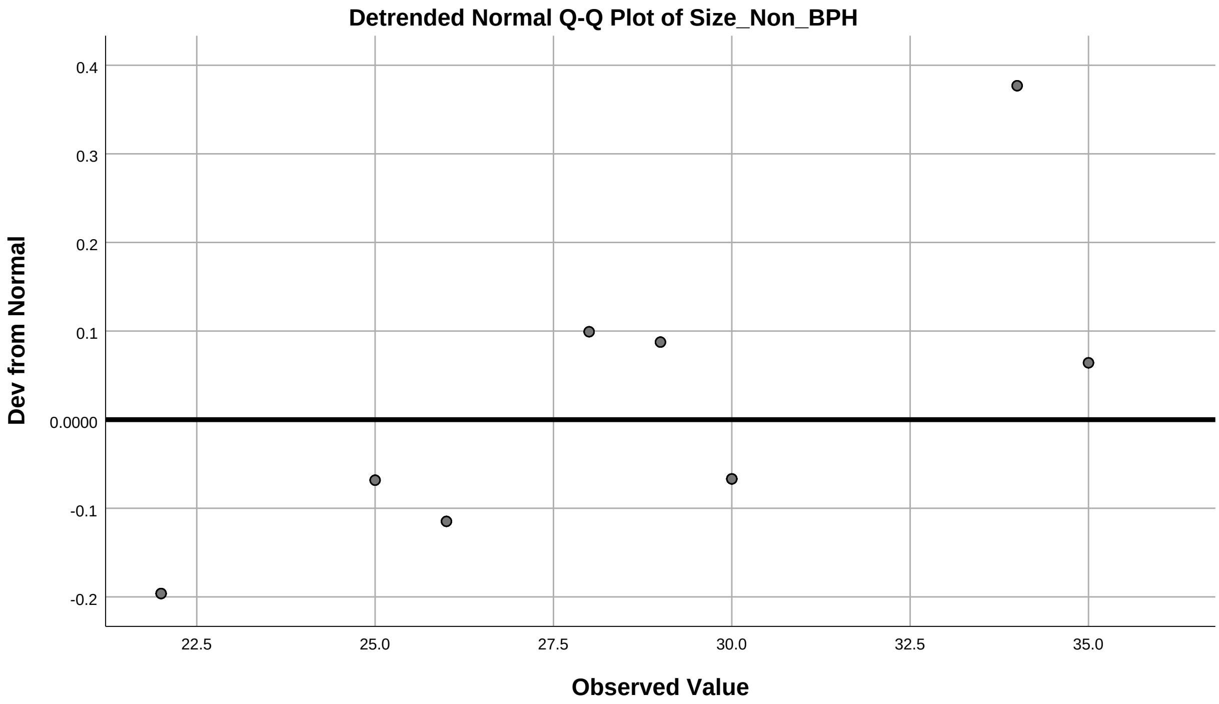

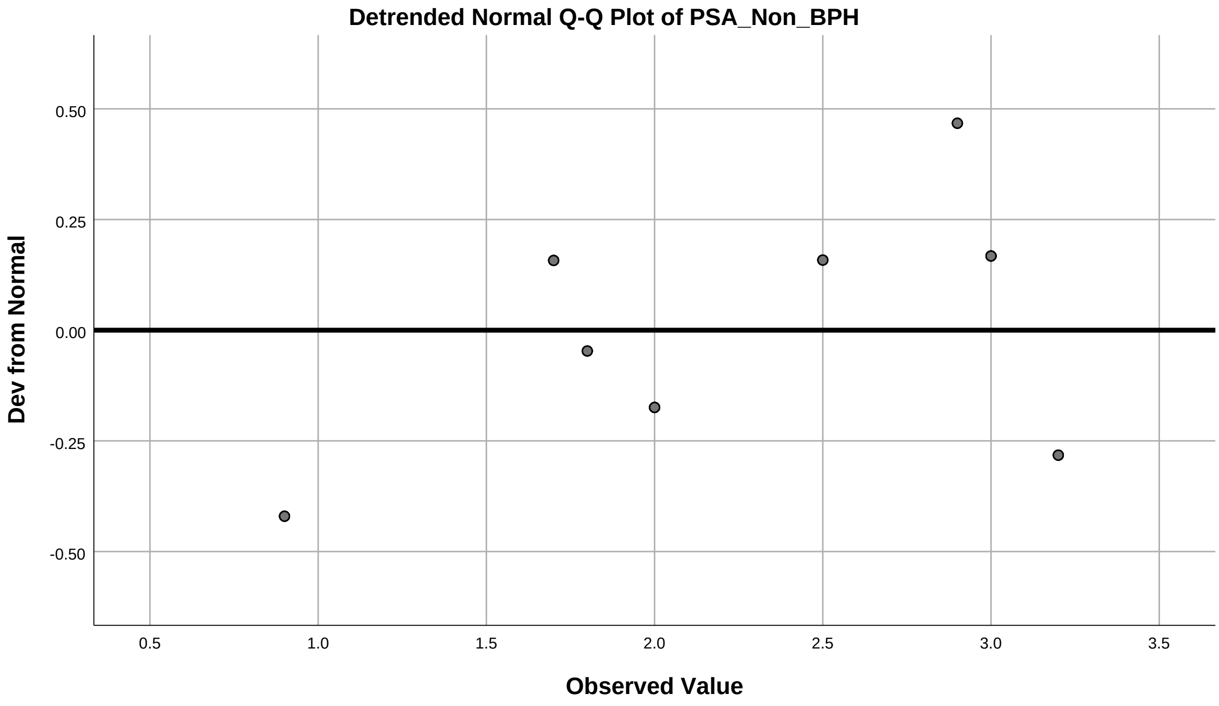

Detrended Normal Q-Q Plot of PSA_Non_BPH
Dev from Normal
0.50
0.25
0.00
-0.25
-0.50
0.5
1.0
1.5
2.0
2.5
3.0
3.5
Observed Value

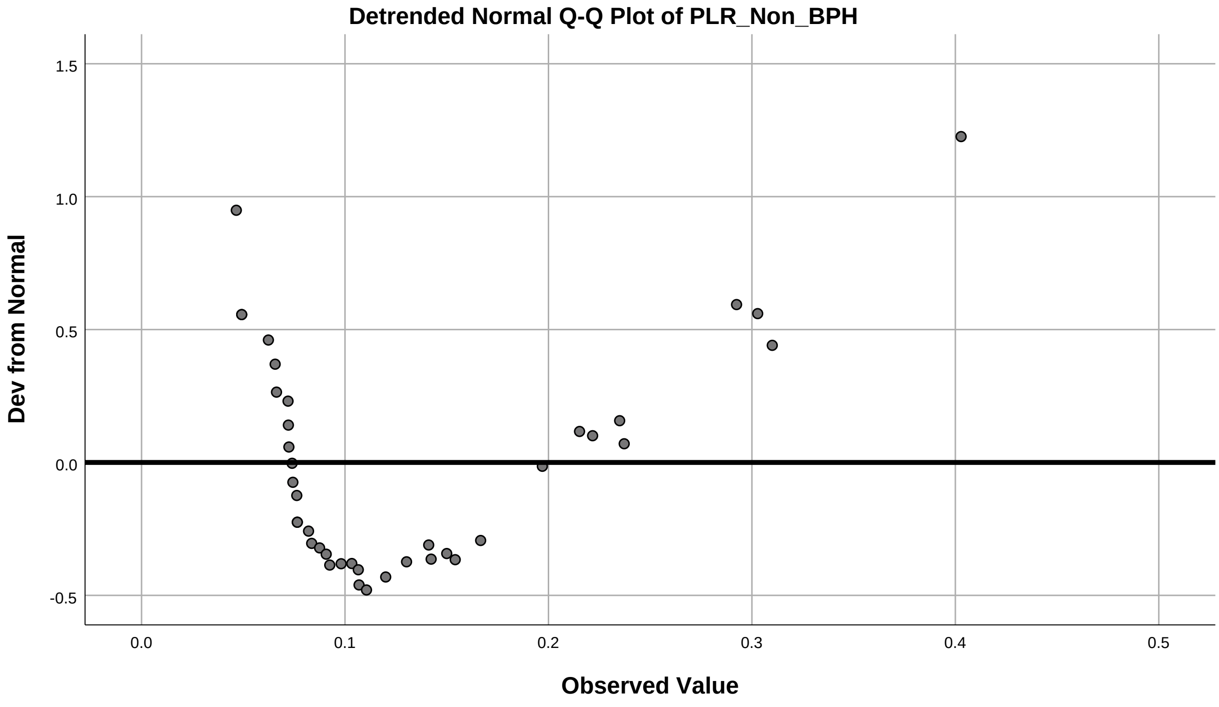

Detrended Normal Q-Q Plot of PLR_Non_BPH
Dev from Normal
1.5
1.0
0.5
0.0
-0.5
0.0
0.1
0.2
0.3
0.4
0.5
Observed Value

Detrended Normal Q-Q Plot of NLR_Non_BPH

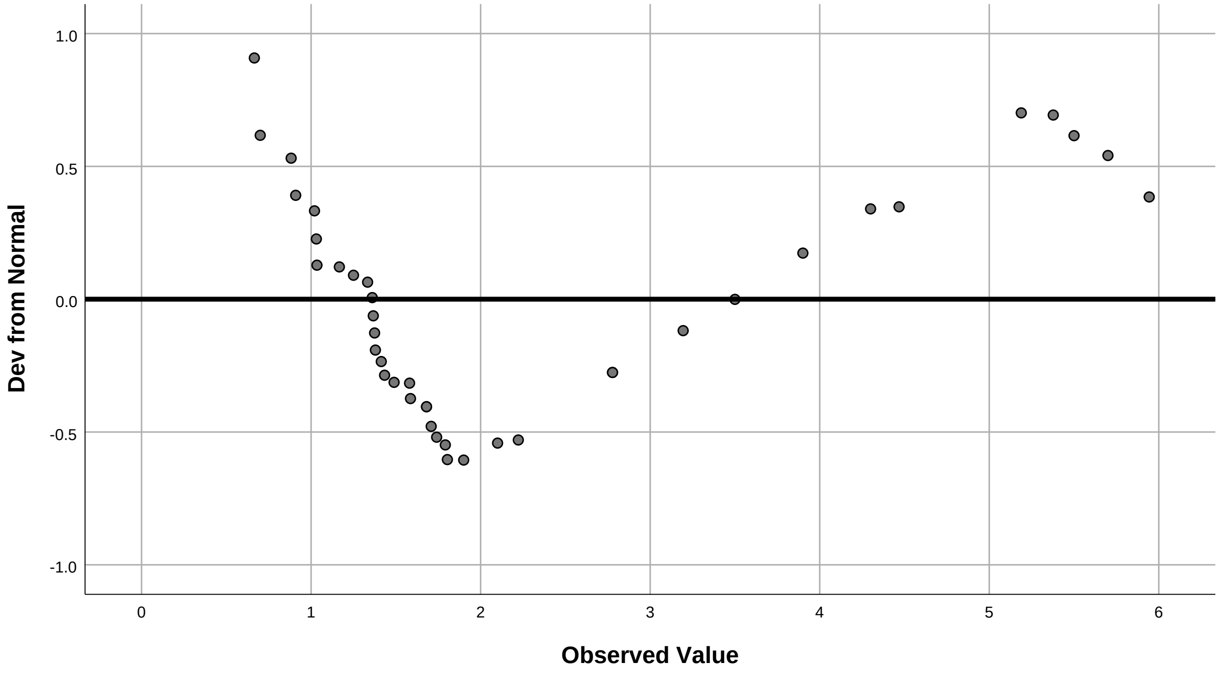

Detrended Normal Q-Q Plot of SII_Non_BPH

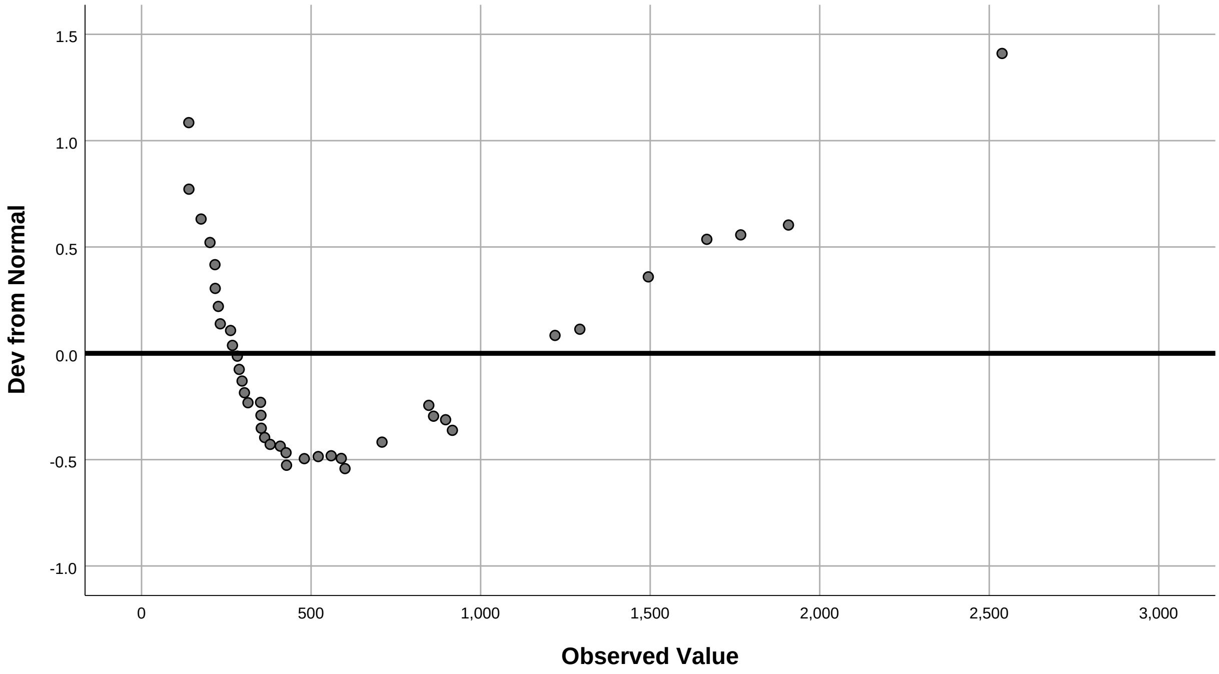

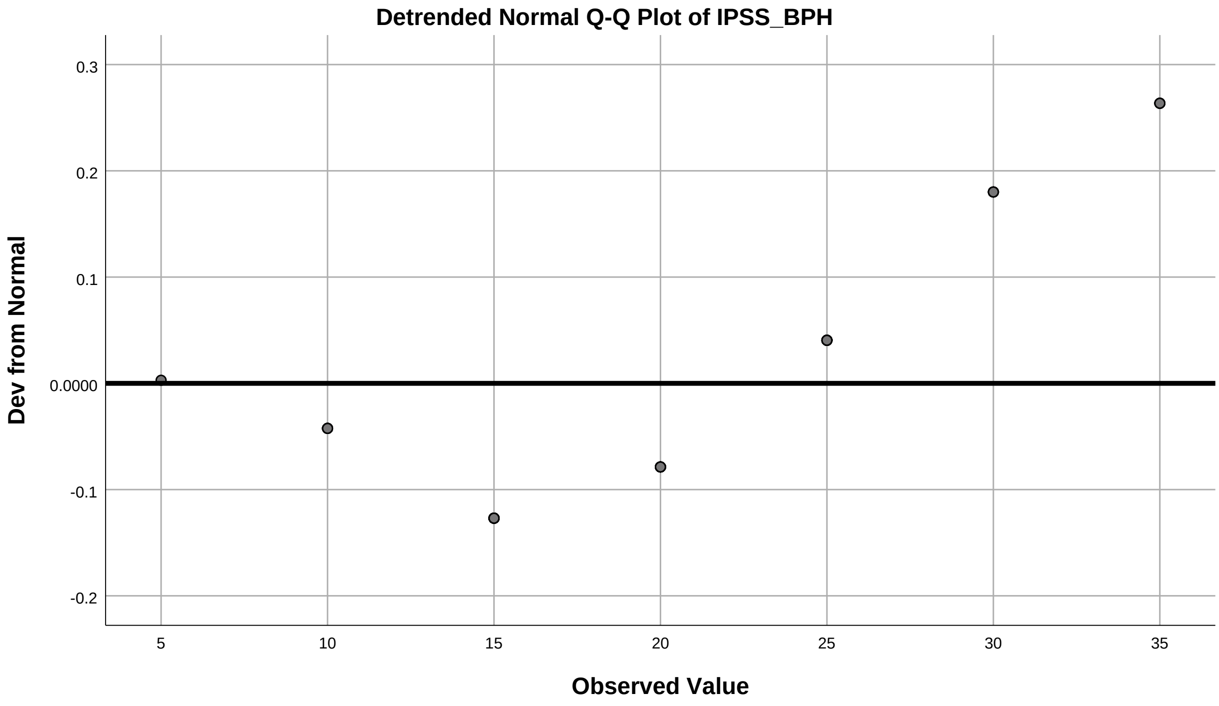
Detrended Normal Q-Q Plot of IPSS_BPH
Dev from Normal
Observed Value
0.3
0.2
0.1
0.0000
-0.1
-0.2
5
10
15
20
25
30
35

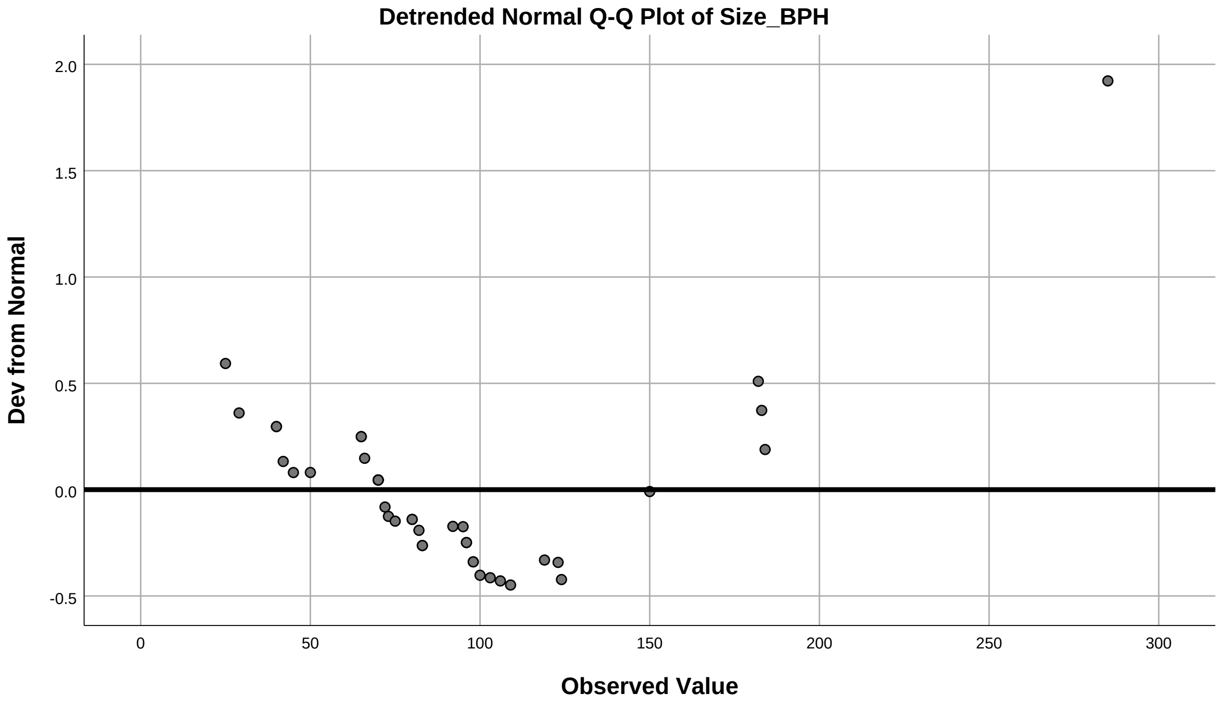
Detrended Normal Q-Q Plot of Size_BPH
Dev from Normal
Observed Value
2.0
1.5
1.0
0.5
0.0
-0.5
0
50
100
150
200
250
300

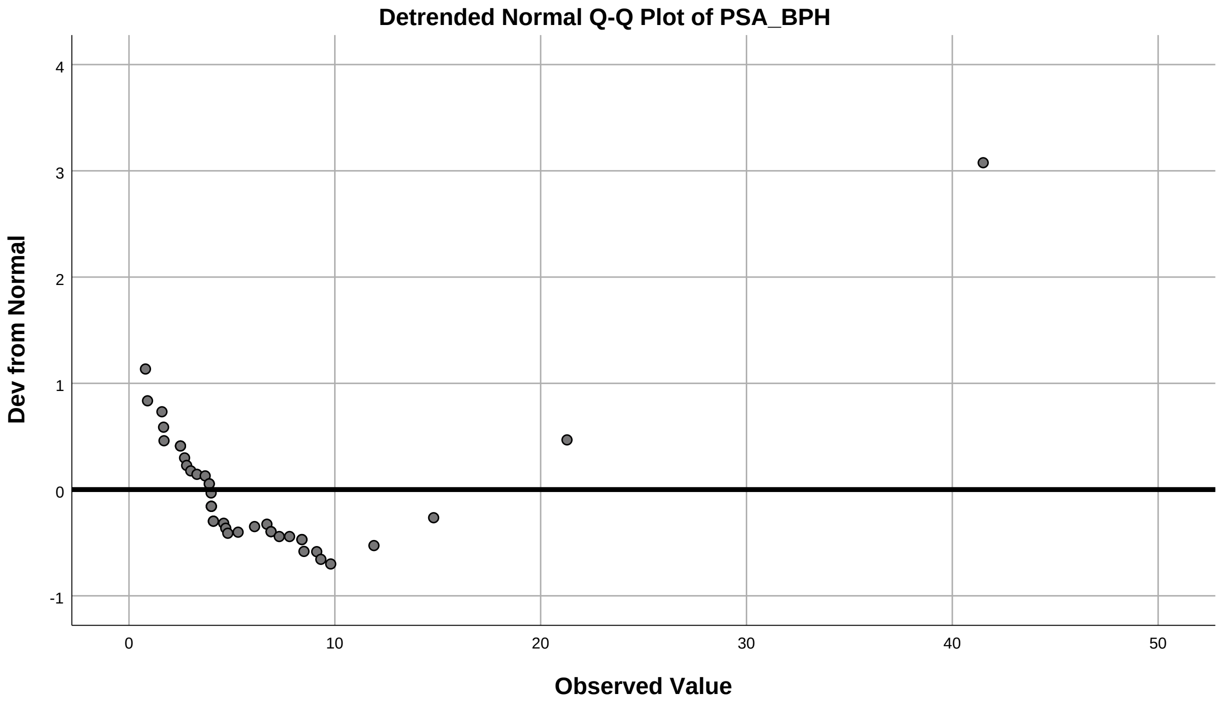
Detrended Normal Q-Q Plot of PSA_BPH
Dev from Normal
4
3
2
1
0
-1
0
10
20
30
40
50
Observed Value

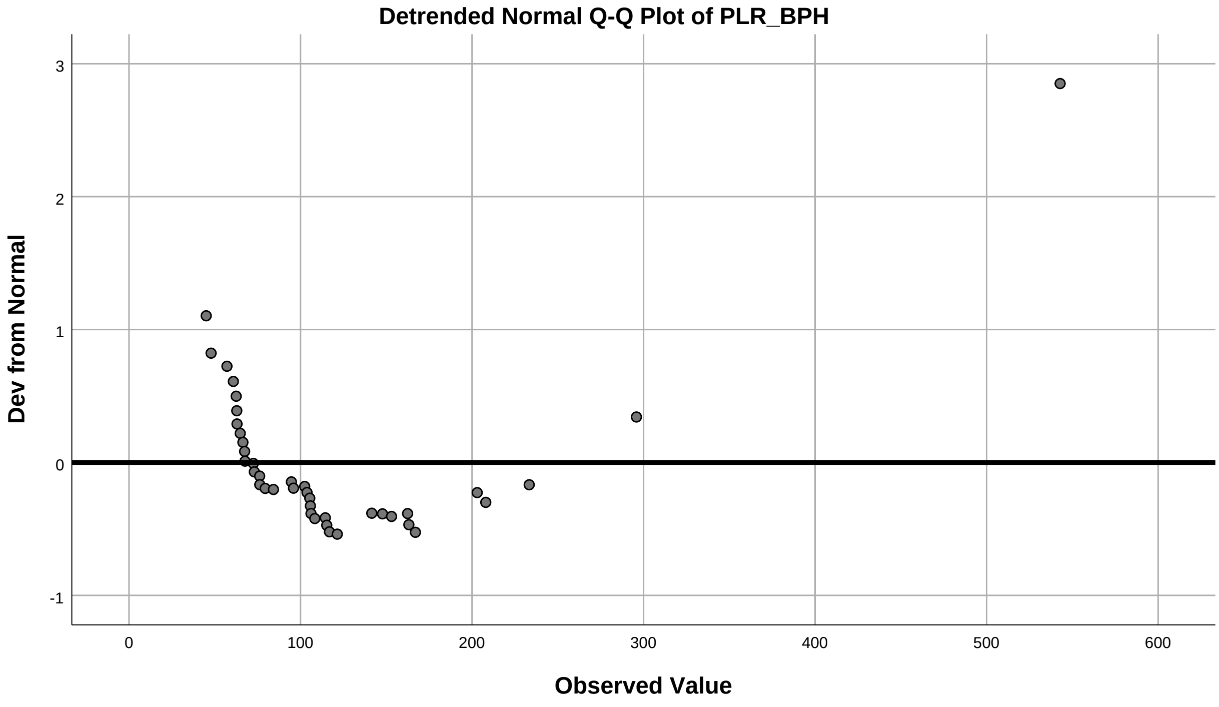
Detrended Normal Q-Q Plot of PLR_BPH
Dev from Normal
3
2
1
0
-1
0
100
200
300
400
500
600
Observed Value

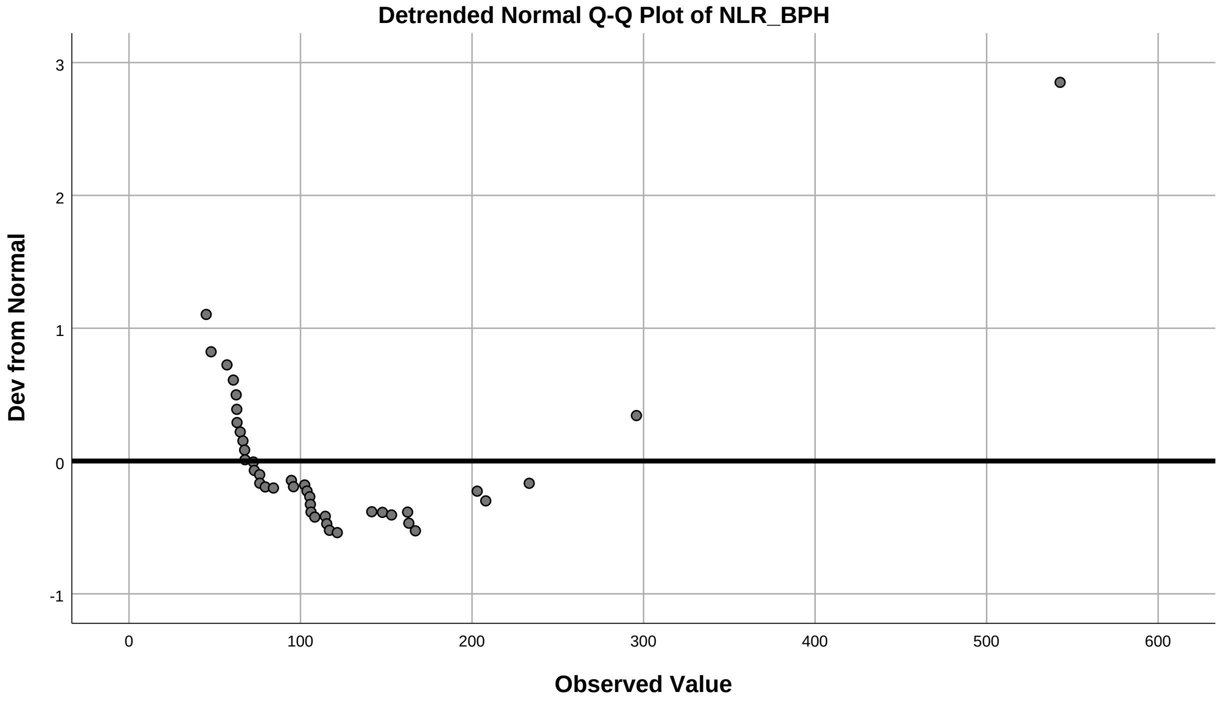
Detrended Normal Q-Q Plot of NLR_BPH
Dev from Normal
Observed Value
3
2
1
0
-1
0
100
200
300
400
500
600

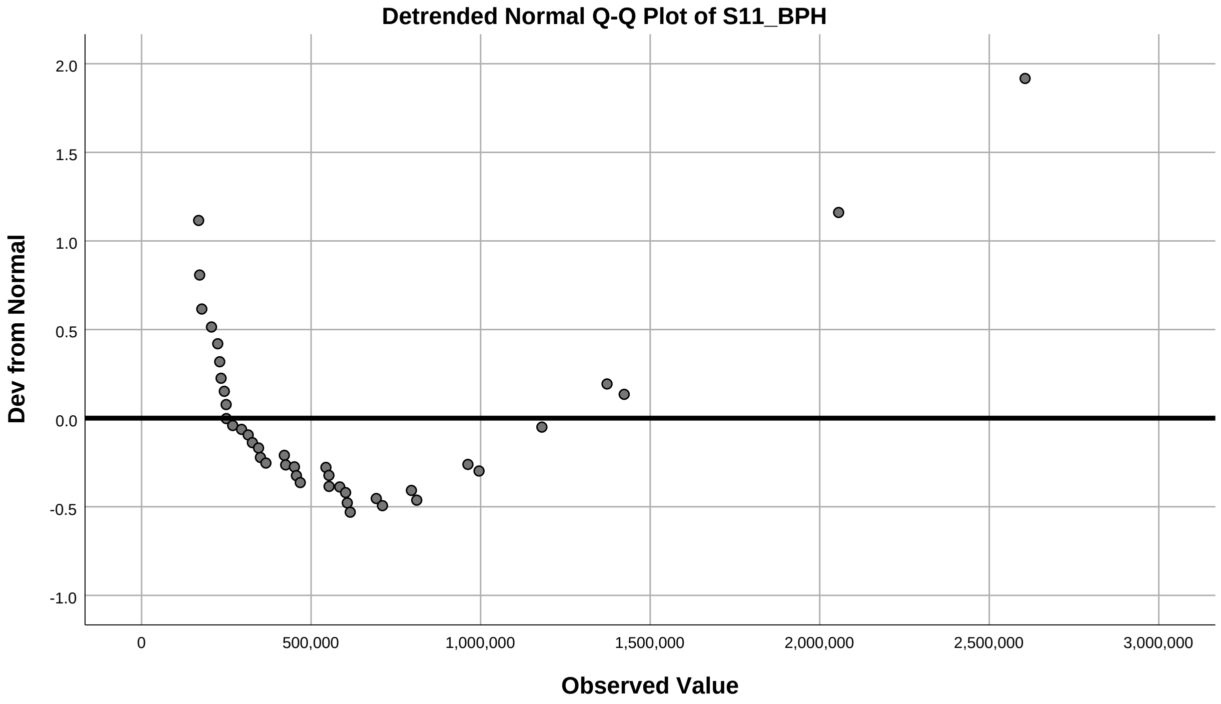
Detrended Normal Q-Q Plot of S11_BPH
Dev from Normal
Observed Value
2.0
1.5
1.0
0.5
0.0
-0.5
-1.0
0
500,000
1,000,000
1,500,000
2,000,000
2,500,000
3,000,000

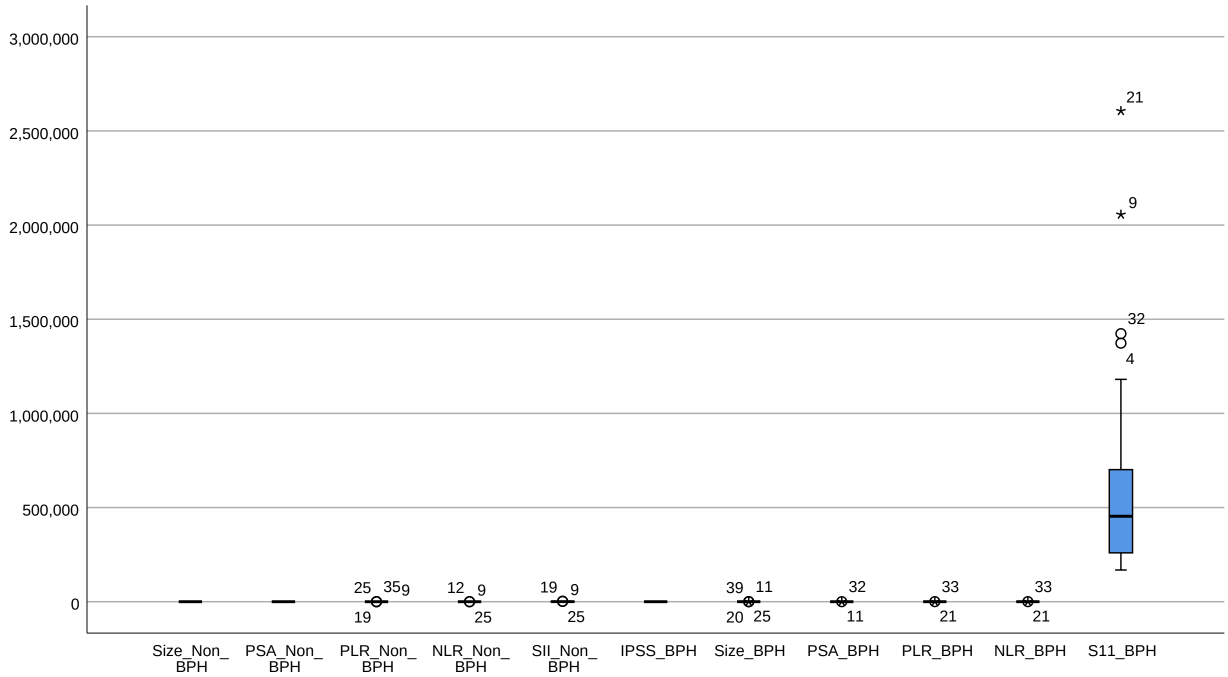
3,000,000
2,500,000
2,000,000
1,500,000
1,000,000
500,000
0
21
9
32
4
25
35
9
19
12
9
25
19
9
25
39
11
20
25
32
11
33
21
33
21
Size_Non_BPH
PSA_Non_BPH
PLR_Non_BPH
NLR_Non_BPH
SII_Non_BPH
IPSS_BPH
Size_BPH
PSA_BPH
PLR_BPH
NLR_BPH
S11_BPH
