# Supplementary material for: Diagnostic efficacy of systemic immune-inflammation biomarkers in benign prostatic hyperplasia using receiver operating characteristic and artificial neural network
Source: Sci Rep. 2023 Sep 8;13:14801. doi: 10.1038/s41598-023-41781-3 (PMC10491602; doi:10.1038/s41598-023-41781-3)
Supplement: Supplementary file 2 — Supplementary Information 2. [file 41598_2023_41781_MOESM2_ESM.pdf]

```
T-TEST GROUPS=draft(0 1)
 /MISSING=ANALYSIS
 /VARIABLES=SII PLR NLR IPSS SIZE PSA
 /CRITERIA=CI(.95).
```

T-Test

Notes

Output Created		29-JUL-2023 09:19:56
Comments		
Input	Data	C:\Users\Lenovo\Desktop\Untitled1.sav
	Active Dataset	DataSet1
	Filter	<none>
	Weight	<none>
	Split File	<none>
	N of Rows in Working Data File	80
Missing Value Handling	Definition of Missing	User defined missing values are treated as missing.
	Cases Used	Statistics for each analysis are based on the cases with no missing or out-of-range data for any variable in the analysis.
Syntax		T-TEST GROUPS=draft(0 1) /MISSING=ANALYSIS /VARIABLES=SII PLR NLR IPSS SIZE PSA /CRITERIA=CI(.95).
Resources	Processor Time	00:00:00.02
	Elapsed Time	00:00:00.01

Group Statistics

	draft	N	Mean	Std. Deviation	Std. Error Mean
SII	Non_BPH	40	637.2571	562.20954	88.89313
	BPH	40	607725.9560	513953.1201	81263.12350
PLR	Non_BPH	40	.1329	.08446	.01335
	BPH	40	120.8840	87.51968	13.83808
NLR	Non_BPH	40	2.3058	1.54434	.24418
	BPH	40	120.8840	87.51968	13.83808
IPSS	Non_BPH	40	.0000	.00000	.00000
	BPH	40	20.6250	7.94109	1.25560
SIZE	Non_BPH	40	28.1000	4.28354	.67729
	BPH	40	92.9500	49.33608	7.80072
PSA	Non_BPH	40	2.3000	.71181	.11255
	BPH	40	6.5823	6.91870	1.09394

Independent Samples Test

		Levene's Test for Equality of Variances		t-test for Equality of Means	
		F	Sig.	t	df
SII	Equal variances assumed	33.953	.000	-7.471	78
	Equal variances not assumed			-7.471	39.000
PLR	Equal variances assumed	25.596	.000	-8.726	78
	Equal variances not assumed			-8.726	39.000
NLR	Equal variances assumed	24.491	.000	-8.568	78
	Equal variances not assumed			-8.568	39.024
IPSS	Equal variances assumed	85.667	.000	-16.426	78
	Equal variances not assumed			-16.426	39.000

Independent Samples Test

		t-test for Equality of Means			
		Sig. (2-tailed)	Mean Difference	Std. Error Difference	95% Confidence ... Lower
SII	Equal variances assumed	.000	-607088.699	81263.17212	-768871.247
	Equal variances not assumed	.000	-607088.699	81263.17212	-771458.967
PLR	Equal variances assumed	.000	-120.75117	13.83808	-148.30068
	Equal variances not assumed	.000	-120.75117	13.83808	-148.74133
NLR	Equal variances assumed	.000	-118.57821	13.84023	-146.13199
	Equal variances not assumed	.000	-118.57821	13.84023	-146.57216
IPSS	Equal variances assumed	.000	-20.62500	1.25560	-23.12470
	Equal variances not assumed	.000	-20.62500	1.25560	-23.16468

Independent Samples Test

		t-test for Equality of Means<br>95% Confidence Interval of the ...<br>Upper
SII	Equal variances assumed	-445306.151
	Equal variances not assumed	-442718.431
PLR	Equal variances assumed	-93.20166
	Equal variances not assumed	-92.76101
NLR	Equal variances assumed	-91.02443
	Equal variances not assumed	-90.58426
IPSS	Equal variances assumed	-18.12530
	Equal variances not assumed	-18.08532

Independent Samples Test

		Levene's Test for Equality of Variances		t-test for Equality of Means	
		F	Sig.	t	df
SIZE	Equal variances assumed	28.076	.000	-8.282	78
	Equal variances not assumed			-8.282	39.588
PSA	Equal variances assumed	14.405	.000	-3.894	78
	Equal variances not assumed			-3.894	39.826

Independent Samples Test

		t-test for Equality of Means			
		Sig. (2-tailed)	Mean Difference	Std. Error Difference	95% Confidence ... Lower
SIZE	Equal variances assumed	.000	-64.85000	7.83007	-80.43846
	Equal variances not assumed	.000	-64.85000	7.83007	-80.68029
PSA	Equal variances assumed	.000	-4.28225	1.09972	-6.47162
	Equal variances not assumed	.000	-4.28225	1.09972	-6.50516

Independent Samples Test

		t-test for Equality of Means
		95% Confidence Interval of the ...
		Upper
SIZE	Equal variances assumed	-49.26154
	Equal variances not assumed	-49.01971
PSA	Equal variances assumed	-2.09288
	Equal variances not assumed	-2.05934
